# siRNAs Targeting Mouse-Specific lncRNA AA388235 Induce Human Tumor Cell Pyroptosis/Apoptosis

**DOI:** 10.3389/fonc.2021.662444

**Published:** 2021-06-14

**Authors:** Yan-Ru Chen, Wan-Ying Feng, Yuan-Xiong Cheng, Hao Zhu, Hong-Juan Liu, Yi Gao, Wei-Jie Zhou

**Affiliations:** ^1^ State Key Laboratory of Organ Failure Research, Guangdong Province Key Laboratory of Molecular Tumor Pathology, Department of Pathology, Nanfang Hospital, School of Basic Medical Sciences, Southern Medical University, Guangzhou, China; ^2^ Department of Research and Teaching, Huizhou Municipal Central Hospital, Huizhou, China; ^3^ Department of Respiratory and Critical Care Medicine, The Third Affiliated Hospital, Southern Medical University, Guangzhou, China; ^4^ Department of Bioinformation, School of Basic Medical Sciences, Southern Medical University, Guangzhou, China; ^5^ General Surgery Center, Department of Hepatobiliary Surgery II, Guangdong Provincial, Research Center for Artificial Organ and Tissue Engineering, Guangzhou Clinical Research and Transformation Center for Artificial Liver, Institute of Regenerative Medicine, Zhujiang Hospital, Southern Medical University, Guangzhou, China; ^6^ Microbiome Medicine Center, Zhujiang Hospital, Southern Medical University, Guangzhou, China; ^7^ Bioland Laboratory (Guangzhou Regenerative Medicine and Health Guangdong Laboratory), Guangzhou, China

**Keywords:** siRNAs, lncRNAs, pyroptosis, apoptosis, tumor

## Abstract

Species-specific lncRNAs significantly determine species-specific functions through various ways, such as epigenetic regulation. However, there has been no study focusing on the role of species-specific lncRNAs in other species yet. Here, we found that siRNAs targeting mouse-specific lncRNA AA388235 could significantly induce death of human tumor cells, although it has no effect on mouse tumor cells and normal human cells. The mechanism studies showed that these siRNAs could activate the response of human tumor cells to exogenous nucleic acids, induce pyroptosis and apoptosis in the presence of GSDME, but induce apoptosis in the absence of GSDME. They also significantly inhibited the growth of human tumor cells *in vivo*. 17 siRNAs were designed for seven more mouse-specific lncRNAs selected randomly, among which 12 siRNAs targeting five lncRNAs induced death in human tumor cell. Our study not only demonstrates that the siRNAs designed for knocking down mouse-specific lncRNA AA388235 can be potential tumor therapeutic drugs, but also suggests that non-human species-specific lncRNAs are a huge potential library that can be used to design siRNAs for tumor treatment. Large-scale screening based on this is promising.

## Introduction

The sequencing of the human genome has revealed that only ~1.2% of the human genome encodes for protein-coding genes, yet the large majority of the human genome is transcribed into non-protein-coding RNAs (ncRNAs) (1, 2). Long non-coding RNAs (lncRNAs) represent the largest group of non-coding RNAs produced from the genome. In the most recent GENCODE V30 release, there are 16,193 annotated lncRNAs in the human genome (3). lncRNAs are defined as transcripts longer than 200 nucleotides that are not translated into proteins, although some transcripts annotated as lncRNAs in fact encode for small proteins (4, 5). They comprise intergenic transcripts (lincRNAs), enhancer RNAs (eRNAs), and sense or antisense transcripts of protein-coding genes (3–6). lncRNAs are reported to have diverse functions, including organization of nuclear architecture, transcription regulation in cis or trans, modulation of mRNA stability, translation and post-translational modification (7, 8).

Compared with highly conserved protein-coding RNAs, lncRNAs generally are poorly conserved and many lncRNA genes are clade- and species-specific (3–9). A recent study reports that lncRNAs evolve rapidly, with >70% of lncRNAs having no sequence-similar orthologs in species separated by >50 million years of evolutionary divergence (8). Increasing reports show that species-specific lncRNAs significantly determine species-specific epigenetic regulation (10–12). However, no studies paid attention to the roles of species-specific lncRNAs in other species.

AA388235 is a mouse-specific lncRNA with a length of 3,867 bp, located in chr17:33981491–33985358, mainly expressed in the testis and heart (13). So far, there is no report focusing on its function. First of all, we randomly designed six siRNAs to knock down lncRNA AA388235. Unexpectedly, although these siRNAs had no effect on the death of mouse tumor cells and normal human cells, they caused death of human colorectal tumor cells and breast tumor cells. These siRNAs increased the response of human tumor cells to exogenous nucleic acids, induced pyroptosis and apoptosis in the presence of GSDME (Gasdermin E), but induced apoptosis in the absence of GSDME. In addition, we randomly designed 17 siRNAs for seven more mouse-specific lncRNAs. 12 of the 17 siRNAs (targeting five different lncRNAs) induced death of human colorectal tumor cells, suggesting that species-specific lncRNAs other than human can be used as targets for designing siRNAs for tumor therapy.

## Materials and Methods

### Antibodies and Reagents

The antibodies to detect P-PKR (ab81303, 1:1,000), GSDME (ab215191, 1:1,000), GSDMA (ab230768, 1:500), and GSDMB (ab215729, 1:500) were obtained from Abcam. Antibodies against caspase-3 (9662S, 1:1,000), cleaved-caspase-3 (9664T, 1:500), PARP (9532T, 1:1,000), MAVS (3993T, 1:1,000), and MDA5 (5321T, 1:1,000) were purchased from Cell Signaling Technologies. Antibodies targeting GSDMD (20770-1-AP, 1:1,000), GSDMC (27630-1-AP, 1:1,000), and caspase9 (10380-1-AP, 1:1,000) were obtained from Proteintech. Anti-RIGI (sc-376845, 1:200) and Anti-TLR3 (sc-32232, 1:200) were obtained from Santa Cruz Biotechnology. Anti-a-tubulin (RM2007, 1:10,000) was obtained from Ray. Caspase-3 inhibitors Z-DEVD-FMK (S7312, 40 µM) were purchased from Selleck.

### Cell Culture

Mouse colorectal tumor cell lines MC38 and CT26 were kindly provided by Professor Wei Yang of the Southern Medical University. Human normal intestinal epithelial cells NCM460 were kindly provided by Professor Side Liu of Nanfang Hospital. Human colorectal tumor cell lines HCT116 and DLD1, mouse breast tumor lines 4T1, human breast tumor cell lines MDA-MB-453, and human normal intestinal epithelial cells FHC were obtained from the American Type Culture Collection (ATCC, Philadelphia, PA, USA). MC38, CT26, and MDA-MB-453 cells were cultured in DMEM (GIBCO) supplemented with 10% fetal bovine serum (FBS). FHCs were cultured in DMEM (GIBCO) supplemented with 20% fetal bovine serum (FBS). HCT116, DLD1, NCM460, and 4T1 cells were cultured in RPMI 1640 (GIBCO) supplemented with 10% fetal bovine serum (FBS). All cells were cultured at 37°C in a humidified 5% CO_2_ incubator.

### Plasmids, siRNAs and Transfection

Penter-human-GSDME-FLAG, PCDNA3.1-mouse-Gsdme-FLAG, PCDNA3.1-mouse-AA388235, and relative negative control plasmids were purchased from Vigene (Shan Dong, China). shNC, shAA388235-2 and shAA388235-6 were purchased from Gene (Shang Hai, China). DsDNAs and siRNAs including ssRNA and dsRNA were synthesized from RiboBio (Guangzhou, China), and all the sequences are listed in **Supplementary Table S1**. For siRNA transfection, cells were plated with 40% confluence in six-well plates, and 50 nM siRNA mixed with 5 µl Lipofectamine 3000 (Invitrogen) was added into the cells after 24 h according to the protocol offered by the manufacturer. For siRNA co-transfection with plasmid, cells were plated with 40% confluence in six-well plates; 50 nM siRNA and 2  µg plasmids mixed with the mixture of 5 µl Lipofectamine 3000 and 10 µl P3000 (Invitrogen) were added into the cells after 24 h. For Z-DEVD treatment, after transfection of the siRNA mixture, 40 µM Z-DEVD was added immediately.

### Microscopy Images

Cells were seeded in the six-well plate format (NEST, China) and treated as indicated. Then static bright-field cell images were captured using an Olympus CKX31 microscope at room temperature. The pictures shown in the figures were processed using ImageJ software. Then the dead cells were washed off with PBS, and the living cells were captured to measure the area percentage of survival cells using Image J software from five independent perspectives.

### SiAA388235-2/4/5/6 Off-Target Prediction

The prediction of siAA388235-2/4/5/6 off-target gene was mainly based on the complementary pairing of siAA388235-2/4/5/6 and the gene, which referred to miRNA. Different software had different algorithms for predicting target genes, and we mainly predicted the off-target gene of siAA388235-2/4/5/6 through four databases: TargetScan, miRanda, RNAhybrid, and pita. We further screened and sorted out the prediction results of the four databases or the random three databases, and the same target genes presented in four or three online databases were considered as potential targets of siAA388235-2/4/5/6.

### RNA Extraction and Quantitative Real-Time PCR

For the Quantitative Real-Time PCR assay, total RNA from cell lines was extracted by Trizol (Takara, Japan) and then reverse-transcribed into cDNA using M-MLV reverse transcriptase (Takara, Japan). qRT–PCR analysis was performed on Applied Biosystems 7500 Fast Real-Time PCR System with the SYBR Premix Ex Taq (Takara, Japan). Primers used for qRT–PCR analysis are listed in **Supplementary Table S2**. All data were using endogenous GAPDH as an internal control.

### Western Blot

Cells were lysed in lysis solution (P0013, Beyotime, China) buffer supplemented with PMSF and under ultrasonic treatment before being boiled in 5× SDS loading buffer (WB-0091, Dingguo, China). Equal amounts of proteins were separated in 10% gradient SDS-PAGE and then electroblotted onto polyvinylidene fluoride (PVDF) membranes, which were then blocked with 5% milk in 0.1% TBST buffer for 1 h before incubation with primary antibodies at 4°C overnight. After washing with TBST 5 min each time for five times, the polyvinylidene fluoride membrane was incubated with appropriate secondary antibody for 1 h at room temperature. The bands were detected using the enhanced chemiluminescence detection system (Amersham Biosciences Europe, Freiberg, Germany).

### LDH Release Assay

Culture supernatants were collected and centrifuged at 250×g for 5 min after treatments. The LDH concentration was measured with the CytoTox 96 cytotoxicity assay (G1780, Promega, USA) at 490 nm according to the protocol offered by the manufacturer. The percentage of LDH release was calculated as follows: (Sample  −  Background)/(Maximum − Background)  ×  100%. Each subject was repeated at least three times.

### ATP Cell Viability

To evaluate the overall cell death, we used the CellTiter-Glo kit (G7570, Promega, USA) to assess cell viability by measuring ATP level. The substrate luciferin and the enzyme luciferase were added into lysed cells to attach to the ATP; the ATP level of treated cells was normalized to the control cells.

### FACS Analysis

Dead and living cells were collected, washed with cold PBS twice, and stained with the FITC-labeled Annexin V and PI (propidium iodide) according to the manufacturer’s recommendation (KGA107, KeyGEN BioTECH, China). After staining, data would be collected by BD FACSCanto II (BD Biosciences) and analyzed by FlowJo V.10 (TreeStar) software. Cell death was assessed by counting Annexin V positive cells. The percentage of Annexin^+^ cells is the sum of PI^+^/Annexin V^+^ and PI^−^/Annexin V^+^ cells.

### Xenograft Mouse Model

Female BALB/c nude mice (4 weeks old) were purchased from the Animal Center of Southern Medical University, Guangzhou, China (NO: 2019038). All the mice used in this study were housed under the specific pathogen-free environment, and animal experiments were conducted in accordance with standard procedures and approved by the Institutional Use Committee for Animal Care. All mice were maintained in a sterile environment. Cells HCT116-shNC (1 × 10^6^), HCT116-shAA388235-2 (1 × 10^6^), and HCT116-shAA388235-6 (1 × 10^6^) were injected into the right flank of the nude mice. Measurement of tumors started 4 days after injection with one measurement every three days. 40 days after injection, the mice were sacrificed; the tumors were excised, weighed, and photographed.

### Statistical Analysis

Data were calculated using SPSS 21.0 (SPSS Inc., Chicago, IL, USA) and GraphPad Prism 6.0 (GraphPad Software Inc., San Diego, CA, USA) then were statistically analyzed by Student’s t-test or analysis of variance (ANOVA). In all cases, data from at least three independent experiments are represented as the mean ± standard error of the mean (SEM). P <0.05 was considered to be statistically significant.

## Results

### siRNAs Targeting Mouse-Specific lncRNA AA388235 Induce Human Tumor Cells Death

**Figure 1:**
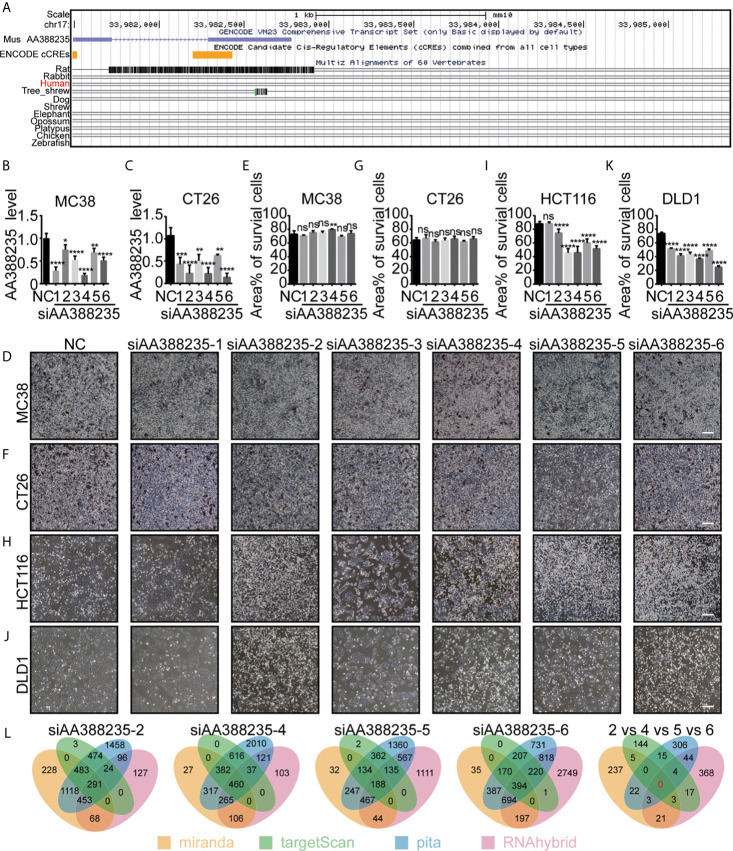
siRNAs targeting mouse-specific lncRNA AA388235 induce human colorectal tumor cells death. **(A)** Graphical views showing multiple-species conversation comparisons with mouse LncRNA AA388235 using UCSC genome browser. The conservation scores were indicated by the gray peaks. **(B, C)** ”NC” was a random arrangement and meaningless 19 bp siRNA, which was desired by RiboBio to act as the negative control.”siAA388235-1/2/3/4/5/6”meant six 19 bp siRNAs targeting six different regions of mouse LncRNA AA388235. The AA388235 level in MC38 and CT26 cells transfected with siRNAs as indicated. *P* value was calculated by one-way ANOVA. **(D–G)** MC38 and CT26 were seeded and treated as indicated. After 48 h, the static bright-field cell images were captured **(D, F)**. Then the dead cells were washed off with PBS, and the living cells were captured to measure the area percentage of survival cells using Image J software from five independent perspectives **(E, G)**. Scale bar, 200μm. *P* value was calculated by one-way ANOVA. **(H–K)** HCT116 and DLD1 were seeded and treated as indicated. After 48 h, the static bright-field cell images were captured **(H, J)**. Then the dead cells were washed off with PBS, and the living cells were captured to measure the area percentage of survival cells using Image J software from five independent perspectives **(I, K)**. Scale bar, 200μm. *P* value was calculated by one-way ANOVA. **(L)** Prediction of off-target genes of siAA388235 using database: miranda, targetScan, pita, and RNAhybrid. “2 *vs* 4 *vs* 5 *vs* 6” meant the prediction of common off-target genes between the siAA388235-2,4,5, and 6. There were no common off-target genes between the siAA388235-2,4,5, and 6 in four databases and ten off-target genes in random three databases including AHRR, C3ORF14, SLC15A2, COQ8A, FAM46C, GOLGA8H, TNRC6C, TLK2,PAGR1, PGS1. Mean ± SEM, *P < 0.05, **P < 0.01, ***P < 0.001, ****P < 0.0001, “ns” indicates no significance.

LncRNA AA388235 is low conserved among species and expressed in mouse but not human (**Figure 1A**). So far, no studies have explored the function of lncRNA AA388235. We first randomly designed six siRNAs targeting mouse lncRNA AA388235. Then, we found that these siRNAs could knocked down the expression of lncRNA AA388235 in mouse colorectal tumor cell lines MC38 and CT26 cells (**Figures 1B, C** **)**. However, they had no effect on the death of MC38 and CT26 cells (**Figures 1D–G** **)**. Neither did they have any effect on the death of mouse breast tumor cells 4T1 (**Supplementary Figures S1A, B**). Surprisingly, four of the six siRNAs targeting mouse lncRNA AA388235 (siAA388235-2, siAA388235-4, siAA388235-5, and siAA388235-6) induced death in human colorectal tumor cell line HCT116 and DLD1; siAA388235-1 had no effect on HCT116 but seemed to inhibit the growth of DLD1 due to fewer survival cells without shedding cells in the phenotype, and siAA388235-3 also seemed to inhibit the growth of HCT116 and DLD1 cells due to fewer survival cells without shedding cells in the phenotype (**Figures 1H–K** and **Supplementary Figures S1C–F**).SiAA388235-2, siAA388235-4, siAA388235-5, and siAA388235-6 also showed death activity in human breast tumor cell line MDA-MB-453 (**Supplementary Figures S1G, H**). However, siAA388235-1/2/3/4/5/6 did not induce death in FHC (human normal intestinal epithelial cells), MCF12A (human normal breast epithelial cells), and HEK293 (human embryonic kidney epithelial cells). In addition, some siRNAs increased the growth of MCF12A and HEK293 cells for unknown reasons (**Supplementary Figures S1I–N**). No death was also observed with the transfection of siAA388235-1/3/4/5/6 into NCM460 (human normal intestinal epithelial cells) except the siAA388235-2 which induced slight death (**Supplementary Figures S1O, P**).

Since lncRNA AA388235 is absent in human cells, we first suspected that the cell death was due to off-target effects of these siRNAs. Miranda, targetscan, pita, and rnahybrid databases were applied to predict the off-target genes of these siRNAs.The results showed that there was no common target gene recognized by four databases among siAA388235-2, siAA388235-4, siAA388235-5, and siAA388235-6, and that 10 genes were recognized by three random databases including AHRR, C3orf14, SLC15A2, COQ8A, FAM46C, GOLGA8H, TNRC6C, TLK2, PAGR1, PGS1 (**Figure 1L**). However, using qPCR assay, we found that there was no correlation between the mRNA level of these ten genes and the four siRNAs targeting mouse lncRNA AA388235 (siAA388235-2, siAA388235-4, siAA388235-5, and siAA388235-6), which further indicated that the death activity caused by siRNAs targeting mouse lncRNA AA388235 was not because they happened to target one common human gene (**Supplementary Figure S2**).

### siRNAs Targeting Mouse-Specific lncRNA AA388235 Induce Human Tumor Cells Pyroptosis or Apoptosis

**Figure 2:**
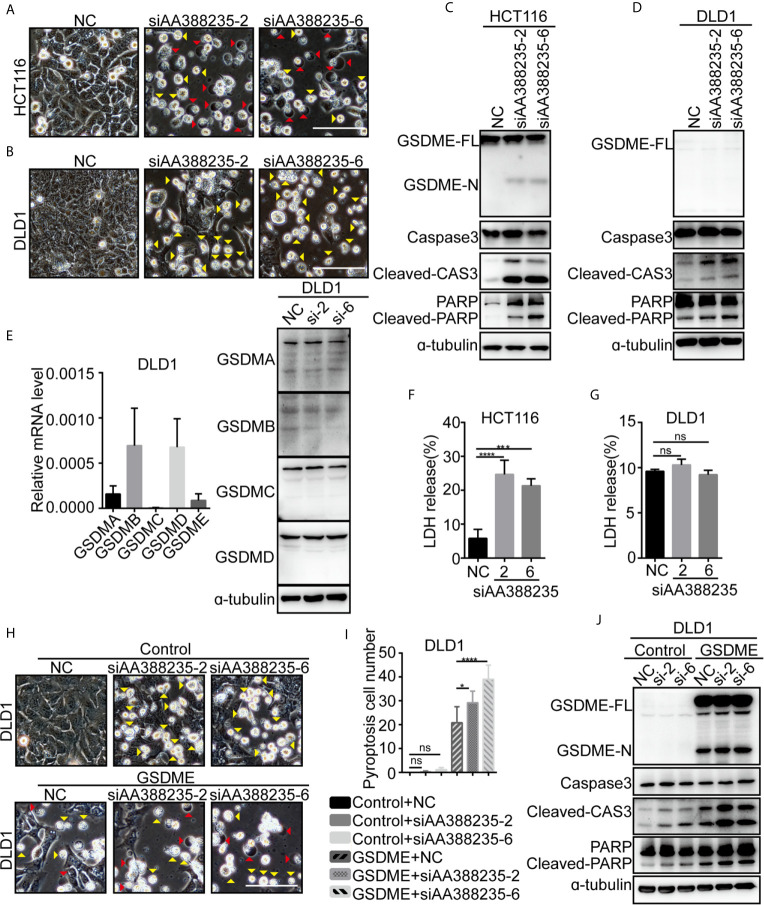
siRNAs targeting mouse-specific lncRNA AA388235 induce human colorectal tumor cells pyroptosis or apoptosis. **(A, B)** Microscopic images of HCT116 and DLD1 cells transfected with siRNAs as indicated. Red arrowheads indicated the pyroptotic cells, and yellow arrowheads indicated the apoptosis cells. Scale bar, 100 μm. **(C, D)** Immunoblotting assay of HCT116 and DLD1 cells transfected as indicated. GSDME-FL, full-length of GSDME; GSDME-N, the N-terminal cleavage of GSDME; Cleaved-CAS3, the cleavage of caspase-3 p19/p17; cleaved-PARP, the cleavage of PARP. **(E)** The mRNA level of GSDM families in wild-type DLD1 cells and the protein level of GSDM families in DLD1 cells transfected with siRNA as indicated. si-2, siAA388235-2; si-6, siAA388235-6. **(F, G)** Comparison of LDH release-based cell death in HCT116 and DLD1 cells transfected with the siRNAs as indicated. *P* value was calculated by one-way ANOVA. **(H)** Microscopic images of DLD1 cells co-transfected with GSDME and siRNA as indicated. Red arrowheads indicate the pyroptotic cells, and yellow arrowheads indicate the apoptosis cells. Scale bar, 100 μm. **(I)** The numbers of the pyroptotic cells of DLD1 co-transfected with GSDME and siRNAs as indicated were calculated in randomly select five fields of view (×200). *P* value was calculated by one-way ANOVA. **(J)** Immunoblotting assay of DLD1 cells co-transfected with GSDME and siRNAs as indicated. GSDME-FL, full-length of GSDME; GSDME-N, the N-terminal cleavage of GSDME; Cleaved-CAS3, the cleavage of Caspase3 p19/p17; Cleaved-PARP, the cleavage of PARP. *P* value was calculated by one-way ANOVA. si-2, siAA388235-2; si-6, siAA388235-6. Mean ± SEM, *P < 0.05, ***P < 0.001, ****P < 0.0001, “ns” indicates no significance.

Next, we explored the mechanism how siRNAs targeting mouse-specific lncRNA AA388235 induce human colorectal tumor cells’ death. siRNAs No. 2 (siAA388235-2) and No. 6 (siAA388235-6) of the six siRNAs were used for the following experiments. We found that the dying HCT116 cells showed evident swelling with characteristic large bubbles from the plasma membrane indicating pyroptosis and shrank and agglomerated indicating apoptosis (**Figure 2A**). Whereas the dying DLD1 cells shrank and agglomerated indicating apoptosis (**Figure 2B**). Recently,Gasdermin E (GSDME) was reported to be cleaved and activated by caspase-3 to cause pyroptosis; therefore it’s expression determined the form of death in caspase-3-activated cells (14, 15). In this study, we found that GSDME was expressed in HCT116 cells and was cleaved upon siRNAs targeting mouse-specific lncRNA AA388235 stimulation (**Figure 2C**), whereas DLD1 cells did not express GSDME with or without siRNA treatment (**Figure 2D**). The other gasdermins (GSDMA, GSDMB, GSDMC, GSDMD) were not expressed or cleaved in DLD1 cells either (**Figure 2E**). The nature of cell death was further confirmed by a lactate dehydrogenase (LDH)-release assay (**Figures 2F, G** **)**. Moreover, overexpression of GSDME in DLD1 cells caused an apoptosis-to-pyroptosis switch upon siAA388235-2 and siAA388235-6 stimulation (**Figures 2H–J**). Similarly, human breast tumor cell line MDA-MB-453, which expressed a high level of GSDME, processed pyroptosis and apoptosis (**Supplementary Figures S3A–C**). However, siAA388235-2 and siAA388235-6 failed to induce pyroptosis or apoptosis in normal human cell lines FHC, MCF12A, and HEK293, regardless of whether the cells expressed GSDME or not (**Supplementary Figures S3D–I**). siAA388235-2 induced slight pyroptosis/apoptosis and the cleavage of GSDME/caspase-3 in NCM460, but siAA388235-6 had no such effect (**Supplementary Figures S3J, K**).

Meanwhile, pyroptosis/apoptosis induced by siAA388235-2 or siAA388235-6 was not observed in mouse colorectal tumor cell lines MC38 and CT26, regardless of whether the cells expressed Gsdme or not (**Supplementary Figures S4A–D**). Overexpression of Gsdme induced MC38 cells pyroptosis, but the cells did not respond to siAA388235-2 and siAA388235-6 (**Supplementary Figures S4E–G**). These results demonstrated that siRNAs targeting mouse-specific lncRNA AA388235 induced human tumor cell pyroptosis and apoptosis in the presence of GSDME, whereas induced human tumor cell apoptosis in the absence of GSDME.

### Caspase-3 Activation Is Necessary for siRNAs Targeting Mouse-Specific lncRNA AA388235 to Induce Human Tumor Cells Pyroptosis or Apoptosis

**Figure 3:**
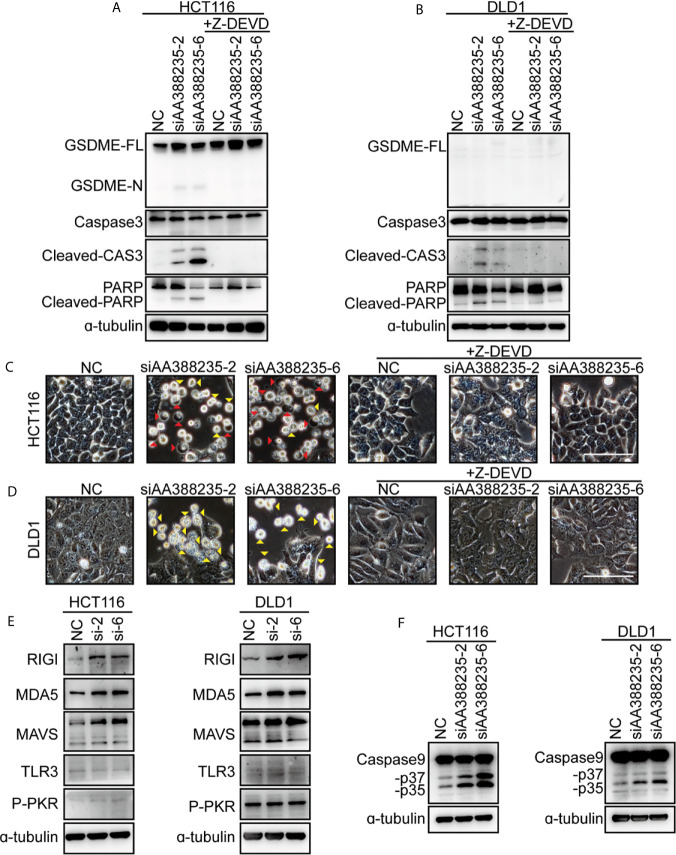
siRNAs targeting mouse-specific lncRNA AA388235 activate caspase-3 and increase RNA sensor. **(A, B)** Effects of Z-DEVD on the cleavage of GSDME and caspase-3 in HCT116 and DLD1 cells transfected with siRNAs as indicated and treated with caspase-3 inhibitor Z-DEVD. GSDME-FL, full-length of GSDME; GSDME-N, the N-terminal cleavage of GSDME; Cleaved-CAS3, the cleavage of caspase-3 p19/p17; cleaved-PARP, the cleavage of PARP. **(C, D)** Effects of Z-DEVD on siRNAs targeting AA388235-induced pyroptosis, and apoptosis in HCT116 and DLD1 cells. Red arrowheads indicated the pyroptotic cells and yellow arrowheads indicated the apoptosis cells. Scale bar, 100 μm. **(E)** Immunoblots of MAVS, RIGI, MDA5, TLR3, and P-PKR (phosphor T451) in HCT116 and DLD1 cells transfected with siRNAs as indicated. si-2, siAA388235-2; si-6, siAA388235-6**. (F)** Immunoblots of caspase-9 in HCT116 and DLD1 cells transfected with siRNAs as indicated. -p37, the cleavage of caspase-9 p37; -p35, the cleavage of caspase-9 p35.

Caspase-3 has long been regarded as the hallmark of apoptosis. Recent studies have shown that GSDME is cleaved and activated by caspase-3, which causes pyroptosis (14, 15). To explore whether caspase-3 activation is necessary for cell pyroptosis or apoptosis induced by siRNAs targeting mouse-specific lncRNA AA388235, caspase-3 inhibitor Z-DEVD was employed. Then, the result showed that caspase-3 cleavage induced by siAA388235-2 and siAA388235-6 was inhibited by Z-DEVD both in HCT116 cells and DLD1 cells (**Figures 3A, B** **)**. Furthermore, both pyroptosis and apoptosis of HCT116 cells and apoptosis of DLD1 cells were inhibited by Z-DEVD (**Figures 3C, D** **)**. These fundings support that caspase-3 activation is necessary for siRNAs targeting mouse-specific lncRNA AA388235 to trigger pyroptosis and apoptosis in human tumor cell.

### siRNAs Targeting Mouse-Specific lncRNA AA388235 Increase RNA Sensor

As siRNAs targeting mouse-specific lncRNA AA388235 are exogenous nucleic acids to human cells, we explored whether they activated host defense pathways. The result showed that the cytosolic double-stranded RNA (dsRNA) sensor RIG-I, MDA5, and MAVS (16, 17) were upregulated upon siAA388235-2 and siAA388235-6 stimulation, whereas the levels of TLR3 (18) and P-PKR did not change (**Figure 3E**). Caspase-9, as the initiator caspase of the intrinsic apoptosis pathway (19), was also activated with the treatment of siAA388235-2 and siAA388235-6 (**Figure 3F**).

### The Form of dsRNA Is Necessary for siRNAs Targeting Mouse-Specific lncRNA AA388235 to Induce Human Tumor Cells Pyroptosis or Apoptosis

**Figure 4:**
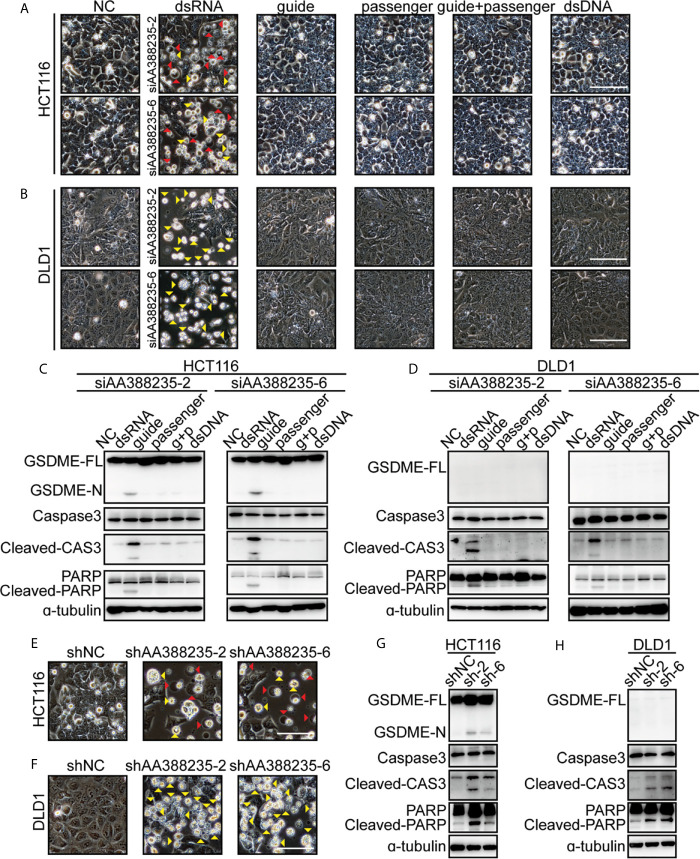
DsRNA is necessary for siRNAs targeting mouse-specific lncRNA AA388235 to induce human tumor cell pyroptosis/apoptosis. **(A, B)** Microscopic images of HCT116 and DLD1 cells transfected as indicated. dsRNA, double-stranded siRNA; guide, single-stranded-anti-sense of siRNA; passenger, single-stranded sense of siRNA; guide + passenger, single-stranded-anti-sense of siRNA and single-stranded-sense of siRNA; dsDNA, double-stranded DNA which has the same sequence as siRNA. Red arrowheads indicated the pyroptotic cells, and yellow arrowheads indicated the apoptosis cells. Scale bar, 100 μm. **(C, D)** Immunoblotting assay of HCT116 and DLD1 cells transfected as indicated. g + p, guide + passenger, single-stranded-anti-sense of siRNA and single-stranded-sense of siRNA. GSDME-FL, full-length of GSDME; GSDME-N, the N-terminal cleavage of GSDME; Cleaved-CAS3, the cleavage of caspase-3 p19/p17; cleaved-PARP, the cleavage of PARP. **(E, F)** Microscopic images of HCT116 and DLD1 cells transfected with shRNA plasmids as indicated. Red arrowheads indicated the pyroptotic cells, and yellow arrowheads indicated the apoptosis cells. Scale bar,100μm. **(G, H)** Immunoblotting assay of HCT116 and DLD1 cells transfected with shRNA plasmids as indicated. sh-2, shAA388235-2; sh-6, shAA388235-6; GSDME-FL, full-length of GSDME; GSDME-N, the N-terminal cleavage of GSDME; Cleaved-CAS3, the cleavage of caspase-3 p19/p17; cleaved-PARP, the cleavage of PARP.

As a double-strand RNA (dsRNA), siRNA consists of two complementary pairs of strands: the strand that determines the specificity of the targeting mRNA serves as the guide RNA, and the other one is a passenger RNA. To test whether the form of dsRNA is necessary for siRNAs targeting mouse-specific lncRNA AA388235 to induce human tumor cells’ pyroptosis or apoptosis, we designed the following experiments. HCT116 cells and DLD1 cells were transfected with dsRNA, single-anti-sense-strand of siRNA (guide), single-sense-strand of siRNA (passenger), single-anti-sense-strand of siRNA plus single-sense-strand of siRNA (guide + passenger) and double-strand DNA (dsDNA), respectively. We found that only dsRNA induced human tumor cells’ pyroptosis and apoptosis (**Figure 4A**) or apoptosis only (**Figure 4B**). Consistently, cleavage of GSDME, caspase-3 and poly ADP-ribose polymerase (PARP) was observed only in HCT116 cells transfected with dsRNA (**Figure 4C**), and cleavage of caspase-3 and PARP was observed only in DLD1 cells transfected with dsRNA (**Figure 4D**). Furthermore, short hairpin RNAs (shRNAs) targeting mouse-specific lncRNA AA388235 also induced pyroptosis and apoptosis in HCT116 cells and apoptosis in DLD1 cells successfully (**Figures 4E–H**). The primary transcript of shRNA contains a hairpin like stem-loop structure, which is transported to the cytoplasm where the loop of the hairpin is processed off to form a double-stranded siRNA (20). These results suggest that the form of dsRNA is necessary for siRNAs targeting mouse-specific lncRNA AA388235 to induce human tumor cells’ pyroptosis or apoptosis.

### shRNAs Targeting Mouse-Specific lncRNA AA388235 Inhibit the Growth of Human Tumor Cells *In Vivo*

**Figure 5:**
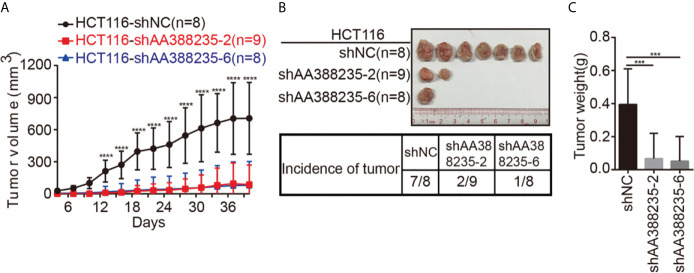
shRNAs targeting mouse-specific lncRNA AA388235 inhibit the growth of human tumor cells *in vivo*. **(A)** Xenograft model in BALB/c nude mice, 1 × 10^6^ cells as indicated were injected to the right flank of nude mice. Measurement of tumor started 4 days after injection and ended 40 days after injection. **(B)** Representative tumor samples from each injected mouse. Only 7/8 mice in the HCT116-shNC group, 2/9 mice in the HCT116-shAA388235-2 group, and 1/8 mice in the HCT116-shAA388235-6 group developed tumors 40 days after injection. **(C)** The weights (g) of tumors in each group as indicated. *P* value was calculated by one-way ANOVA. Mean ± SEM, ***P < 0.001, ****P < 0.0001.

To investigate whether the shRNAs targeting mouse-specific lncRNA AA388235 inhibited the growth of human tumor cells *in vivo*, HCT116 cells transfected with control shRNA, shAA388235-2, or shAA388235-6 were subcutaneously inoculated into BALB/c nude mice. The tumor incidence and tumor size were dramatically reduced by shAA388235-2 or shAA388235-6 (**Figures 5A–C**).

### Multiple siRNAs Targeting Different Mouse-Specific lncRNAs Induce Human Tumor Cells Pyroptosis or Apoptosis

**Figure 6:**
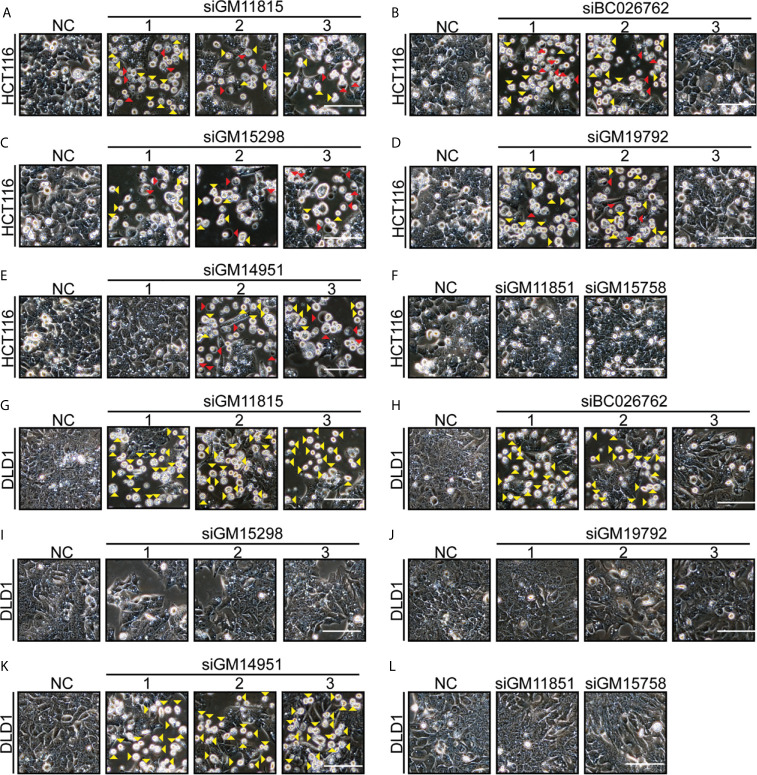
Multiple siRNAs targeting different mouse-specific lncRNAs induce human colorectal tumor cells pyroptosis or apoptosis. Microscopic images of HCT116 and DLD1 cells transfected with siRNAs as indicated. Red arrowheads indicated the pyroptotic cells, and yellow arrowheads indicated the apoptosis cells. Scale bar, 100 μm. **(A)** Transfection with si-GM11815-1/2/3 induced HCT116 cells’ pyroptosis and apoptosis. **(B)** Transfection with si-BC026762-1/2 induced HCT116 cells’ pyroptosis and apoptosis, and transfection with si-BC026762-3 had no effect on HCT116 cells. **(C)** Transfection with si-GM15298-1/2/3 induced HCT116 cells’ pyroptosis and apoptosis. **(D)** Transfection with si-GM19792-1/2 induced HCT116 cells’ pyroptosis and apoptosis, and transfection with si-GM19792-3 had no effect on HCT116 cells. **(E)** Transfection with si-GM14951-1 had no effect on HCT116 cells, and transfection with si-GM14951-2/3 induced HCT116 cells’ pyroptosis and apoptosis. **(F)** Transfection with si-GM11851 and si-GM15758 had no effect on HCT116 cells. **(G)** Transfection with si-GM11815-1/2/3 induced DLD1 cells apoptosis. **(H)** Transfection with si-BC026762-1/2 induced DLD1 cells apoptosis and transfection with si-BC026762-3 had no effect on DLD1 cells. **(I)** Transfection with si-GM15298-1/2/3 inhibited the growth of DLD1 cells. **(J)** Transfection with si-GM19792-1/2/3 had no effect on DLD1 cells. **(K)** Transfection with si-GM14951-1/2/3 induced DLD1 cells apoptosis. **(L)** Transfection with si-GM11851 and si-GM15758 had no effect on DLD1 cells. Scale bar, 100 μm.

There are thousands of mouse-specific lncRNAs. Seven more mouse-specific lncRNAs (GM11815, BC026762, GM15298, GM19792, GM14951, GM11851, and GM15758) were randomly selected. Conservation comparisons using UC Santa Cruz (UCSC) genome browse showed that these seven lncRNAs were not found in humans (**Supplementary Figures S5**). In total, 17 siRNAs were designed for these seven mouse-specific lncRNAs. Next, we found that 12 siRNAs (targeting five different lncRNAs: GM11815, BC026762, GM15298, GM19792, and GM14951) induced HCT116 cells’ pyroptosis and apoptosis (**Figures 6A–F**). Consistently, these 12 siRNAs induced cleavage of GSDME, caspase-3, and PARP (**Supplementary Figures S6A–F**). Eight siRNAs (targeting three different lncRNAs: GM11815, BC026762, and GM14951) induced DLD1 cells’ apoptosis (**Figures 6G–L**). Consistently, these eight siRNAs induced cleavage of caspase-3 and PARP (**Supplementary Figures S6G–L**). Among these siRNAs, siRNAs targeting GM11815, BC026762, and GM14951 had similar functions to those targeting AA388235, which induced pyroptosis and apoptosis in HCT116 cells (**Figures 6A, B, E** **)** and induced apoptosis in DLD1 cells (**Figures 6G, H, K** **)**. Whereas siRNAs targeting GM15298 induced HCT116 cells’ pyroptosis and apoptosis (**Figure 6C**), inhibited DLD1 cells’ growth rather than induced apoptosis (**Figure 6I**). SiRNAs targeting GM19792 induced HCT116 cells’ pyroptosis and apoptosis (**Figure 6D**) and had no effect on DLD1 cells (**Figure 6J**). Meanwhile, siRNAs targeting GM11851 and GM15758 had no effect on HCT116 cells and DLD1 cells (**Figures 6F, L** **)**. These results indicate that siRNAs targeting mouse-specific lncRNAs have cytotoxicity to human tumor cells, which is a broad-spectrum phenomenon.

### lncRNA AA388235 Induces Pyroptosis or Apoptosis of Human Tumor Cells Rather Than Mouse Tumor Cells

**Figure 7:**
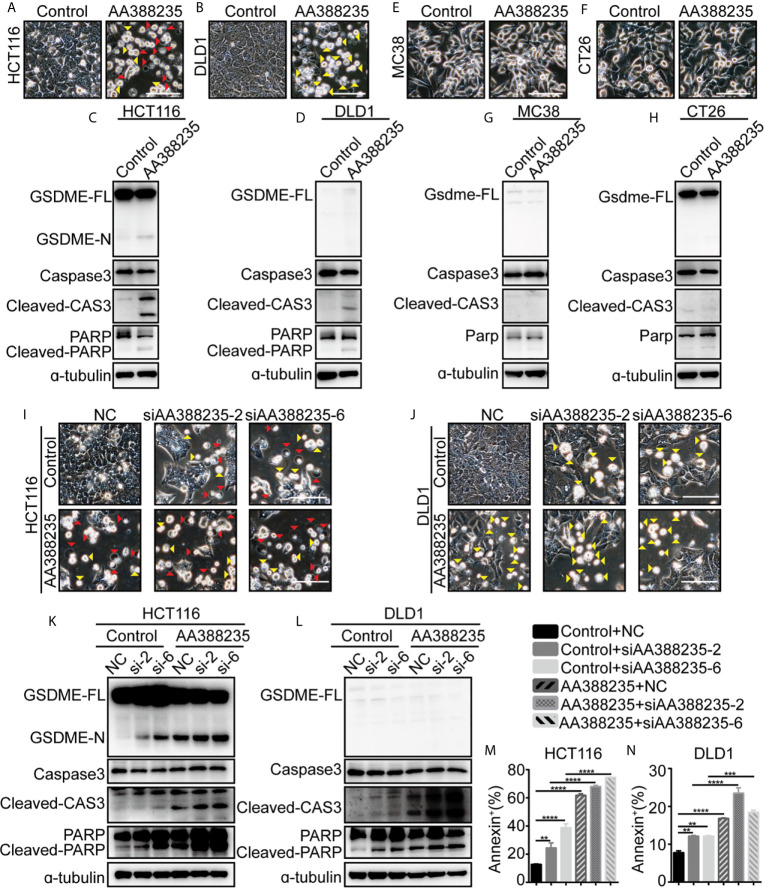
IncRNA AA388235 induces pyroptosis or apoptosis of human tumor cells rather than mouse tumor cells. **(A, B)** Microscopic images of HCT116 and DLD1 cells transfected with plasmids as indicated. Red arrowheads indicated the pyroptotic cells, and yellow arrowheads indicated the apoptosis cells. Scale bar, 100 μm. **(C, D)** Immunoblotting assay of HCT116 and DLD1 cells transfected with plasmids as indicated. GSDME-FL, full-length of GSDME; GSDME-N, the N-terminal cleavage of GSDME; cleaved-CAS3, the cleavage of caspase-3 p19/p17; cleaved-PARP, the cleavage of PARP **(E, F)** Microscopic images of MC38 and CT26 cells transfected with plasmids as indicated. Scale bar, 100 μm. **(G, H)** Immunoblotting assay of MC38 and CT26 cells transfected with plasmid as indicated. Gsdme-FL, full-length of Gsdme; Cleaved-CAS3, the cleavage of caspase-3 p19/p17. **(I, J)** Microscopic images of HCT116 and DLD1 cells co-transfected with control or AA388235 plasmids and siRNAs as indicated. Red arrowheads indicated the pyroptotic cells, and yellow arrowheads indicated the apoptosis cells. Scale bar, 100 μm. **(K, L)** Immunoblotting assay of HCT116 and DLD1 cells co-transfected with control or AA388235 plasmid and siRNA as indicated. si-2, siAA388235-2; si-6, siAA388235-6. GSDME-FL, full-length of GSDME; GSDME-N, the N-terminal cleavage of GSDME; Cleaved-CAS3, the cleavage of caspase-3 p19/p17; cleaved-PARP, the cleavage of PARP. **(M, N)** Flow cytometry of PI (propidium iodide) and Annexin V-fluorescein isothiocyanate (FITC)-stained cells. The percentage of Annexin^+^ cells is the sum of PI^+^/Annexin V^+^ and PI^-^/Annexin V^+^ cells. *P* value was calculated by one-way ANOVA. Mean ± SEM, **P < 0.01, ***P < 0.001, ****P < 0.0001.

We next examined whether lncRNA AA388235 itself affects human tumor cells. Similar to siRNAs targeting lncRNA AA388235, overexpression of lncRNA AA388235 (**Supplementary Figures S7A, B**) induced pyroptosis and apoptosis in HCT116 cells (**Figure 7A**) and apoptosis in DLD1 cells (**Figure 7B**). Consistently, cleavage of GSDME, caspase-3, and PARP was observed in HCT116 cells transfected with AA388235 (**Figure 7C**), whereas only cleavage of caspase-3 and PARP was observed in DLD1 cells transfected with AA388235 (**Figure 7D**). However, overexpression of lncRNA AA388235 (**Supplementary Figures S7C, D**) had no effect on mouse tumor cell lines MC38 and CT26 cells (**Figures 7E–H**). Furthermore, the combination of lncRNA AA388235 and siRNAs targeting lncRNA AA388235 increased cell death compared with siRNAs targeting lncRNA AA388235 alone (**Figures 7I–N** and **Supplementary Figures S7E, F**), which was probably because more fragments were produced during the degradation of AA388235 by siRNAs.

## Discussion

In recent decades, scientists have been searching for reliable targets for tumor treatment from various perspectives. One of the most common research strategies is to target some mutated genes or abnormal signaling pathways. As more cancer genomes are sequenced, a large number of cancer genes were identified which is critical for the development of target-based cancer therapeutics. Therefore, it is of great significance to search for new sources of tumor therapeutic targets. We suggest that species-specific lncRNAs can be used to construct new screening libraries, which was supported by our discoveries from this study. We randomly selected eight mouse-specific lncRNAs (absent in human) and designed 23 siRNAs targeting these eight lncRNAs. Among them, around 12**–**16 siRNAs (targeting six different mouse-specific lncRNAs) induced human tumor cells’ pyroptosis or apoptosis. Among them, the mouse-specific lncRNA AA388235 was used to study the underlying mechanism. We next randomly designed six siRNAs targeting AA388235, four of which caused the death of human tumor cell. It was found that siRNAs targeting AA388235 activated the response of human tumor cells to exogenous nucleic acids then induced pyroptosis and apoptosis in the presence of GSDME, but only apoptosis in the absence of GSDME. By sequence analysis and qPCR, we ruled out the possibility that these siRNAs were targeting a common gene that could cause cell death. The reasonable explanation of these findings is that these siRNAs induce cell death by activating the host cell response to exogenous nucleic acids. Further experiments were performed to verify this explanation, with results showing that RNA sensors RIG-I, MDA5, and MAVS are increased.

Numerous metazoan lncRNAs have been discovered from cDNA libraries and RNA-seq data by high-throughput transcriptome projects. Among them, only a handful of lncRNAs have been functionally characterized. Meanwhile, many lncRNAs do not show the same pattern of high interspecies conservation as protein-coding genes. The lack of functional studies and poor evolutionary conservation have raised concerns of whether non-coding RNAs represent ‘‘transcriptional noise’’ or truly functional biomolecules (20–22). At present, the understanding of the function of lncRNA is only achieved from detailed studies on a case-by-case basis, which only focus on lncRNA function in its expression site. No attention has been paid to the fact that non-conserved lncRNAs may serve as species-specific features, and the possibility that species-specific lncRNAs can be recognized as exogenous nucleic acids by the host. For the first time, our studies showed that mouse-specific lncRNA AA388235 induces human tumor cells’ pyroptosis or apoptosis, although it has no effect on mouse tumor cells. More interestingly, siRNAs targeting lncRNA AA388235 have the same effect as lncRNA AA388235. In addition to AA388235, siRNAs targeting seven other mouse-specific lncRNAs were randomly designed, and multiple siRNAs also induced death in human tumor cell. We hypothesized that the effect of such species-specific lncRNAs might be universal and unveiled a new source of target libraries. As there are abundant lncRNAs specific to other mammals, with many of which having similar functions, a lot of siRNAs targeting these lncRNAs can be designed to build a siRNA library. And we are trying to build an extensive screening siRNA library to find out new anti-tumor drugs.

The discovery of RNA interference (RNAi) is a seminal work (23). Currently, siRNAs have been used as useful tools in biological research to suppress any gene achieved conveniently by a base sequence alone (24, 25). At the same time, siRNAs can be easily chemically synthesized and have the prospect to inhibit the genes encoding proteins that are “undruggable” by classical small molecules (26, 27). This has led many companies to develop the therapeutic potential of siRNAs. However, the clinical trials have shown non-specific toxicity and insufficient efficacy (28–30). Non-specific toxicity causes severe side effects in the development of siRNA therapy, including immunogenic reactions to dsRNA, unintended RNAi activity caused by unintended seed region matches between guide strands and non-targeted mRNAs, and on-target RNAi activity by siRNA drugs that accumulated in non-target tissues (30–32). These challenges make it difficult to utilize siRNA in clinical trial. However, siRNAs targeting non-human species-specific lncRNAs offer new opportunities to overcome these challenges. First, these siRNAs induce human tumor cells death by activating cell responses to exogenous nucleic acids, but do not activate toll-like receptors (TLRs) that activate immune system cells. Second, siRNAs that may activate host immune reactions or are toxic to human normal cells can be excluded by pre-screening. Thus, unintended RNAi activities and accumulation of siRNAs in non-target tissues can be effectively avoided. The large number of species-specific lncRNAs allows many siRNAs to be designed upon their sequences. After pre-screening siRNAs causing immune reactions and toxicity to normal cells, lots of siRNAs may still remain as candidate drugs. In this study, for example, we found that multiple siRNAs targeting mouse-specific lncRNA AA388235 induced human tumor cells death without having toxicity to human normal cells. And clinical trials are expected to test their anti-tumor effect.

More studies are needed to explore the mechanism. Our study shows that both double stranded siRNA and shRNA can induce cell death, while single stranded siRNA or dsDNA cannot. Full-length lncRNA can also induce cell death, which may be due to the formation of special secondary structures. Furthermore, it is unknown why human tumor cells are more sensitive to these siRNA induced death than human normal cells. It was reported that many oncogenic insults deregulated RNA splicing leading to hypersensitivity of tumors to spliceosome targeted therapies (STTs) which caused widespread cytoplasmic accumulation of dsRNA to trigger antiviral signaling and extrinsic apoptosis (33), suggesting that the sensitivity of tumor cells might be due to the abnormally activated oncogenic gene. The detailed molecules and signaling pathways need to be further studied. Moreover, a large proportion of siRNAs (16/23) induced cell death, while some other siRNAs failed, partly because of the special sequence characteristics of these siRNAs. High throughput screening and sequence analysis are considered to help address these issues.

In conclusion, we found that siRNAs targeting mouse-specific lncRNA AA388235 could induce pyroptosis or apoptosis in human tumor cells without toxicity to human normal cells. Through the functional verification of 17 siRNAs designed for the seven other mouse-specific lncRNAs, we propose that species-specific lncRNAs can be used as sequence targets to design siRNAs for tumor treatment, which highlights a novel approach to tumor drug design.

## Data Availability Statement

The original contributions presented in the study are included in the article/**Supplementary Material**. Further inquiries can be directed to the corresponding author.

## Ethics Statement

The animal study was reviewed and approved by Ethics Committee of Southern Medical University, Guangzhou, China (NO:2019038).

## Author Contributions

The project was conceived by W-JZ. The experiments were designed by W-JZ and Y-RC. The cell biology function experiments and animal experiments were performed by Y-RC and W-YF. The statistical analysis was completed by Y-RC, HZ and H-JL. Data were analyzed by W-JZ, Y-RC, YC and YG. The manuscript was written by W-JZ. All authors contributed to the article and approved the submitted version.

## Funding

This work was supported by the National Natural Science Foundation of China (92068206, 81573015 and 81600496), Key-Area Research and Development Program of Guangdong Province (2019B020234003), Frontier Research Program of Bioland Laboratory (Guangzhou Regenerative Medicine and Health Guangdong Laboratory) (2018GZR110105002), Clinical Innovation Research Program of Bioland Laboratory (Guangzhou Regenerative Medicine and Health Guangdong Laboratory) (2018GZR0201003); Outstanding Scholar Program of Bioland Laboratory Guangzhou Regenerative Medicine and Health Guangdong Laboratory) (2018GZR110102004) Funding for open access charge: National Natural Science Foundation of China (92068206).

## Conflict of Interest

The authors declare that the research was conducted in the absence of any commercial or financial relationships that could be construed as a potential conflict of interest.

## Supplementary Material

Supplementary Figure 1 siRNAs targeting mouse-specific lncRNA AA388235 have no/little effect on 4T1 and human normal cells but induce human tumor cells death. **(A, B)** 4T1 cells were seeded and treated as indicated. After 48 h, the static bright-field cell images were captured **(A)**. Then the dead cells were washed off with PBS, and the living cells were captured to measure the area percentage of survival cells using Image J software from five independent perspectives **(B)**.Scale bar, 200μm. *P* value was calculated by one-way ANOVA. **(C, D)** Comparison of ATP cell viability in HCT116 and DLD1 cells transfected with the siRNAs as indicated. *P* value was calculated by one-way ANOVA. **(E, F)** Flow cytometry of PI (propidium iodide) and Annexin V-fluorescein isothiocyanate (FITC)-stained cells. The percentage of Annexin^+^ cells is the sum of PI^+^/Annexin V^+^ and PI^−^/Annexin V^+^ cells. *P* value was calculated by one-way ANOVA. **(G, H)** MDA-MB-453 cells were seeded and treated as indicated. After 48 h, the static bright-field cell images were captured **(G)**. Then the dead cells were washed off with PBS, and the living cells were captured to measure the area percentage of survival cells using Image J software from five independent perspectives **(H)**.Scale bar, 200μm. *P* value was calculated by one-way ANOVA. **(I–P)** Cells were seeded and treated as indicated. After 48 h, the static bright-field cell images were captured **(I, K, M, O)**. Then the dead cells were washed off with PBS, and the living cells were captured to measure the area percentage of survival cells using Image J software from five independent perspectives **(J, L, N, P)**. Scale bar, 200μm. *P* value was calculated by one-way ANOVA. Mean ± SEM, **P* < 0.05, ***P* < 0.01, ****P* < 0.001, *****P* < 0.0001, “ns” indicates no significance.

Supplementary Figure 2 siRNAs targeting mouse-specific lncRNA AA388235 do not target one common human gene. **(A, B)** The detection of predicted common off-target gene between the siAA388235-2,4,5, and 6 recognized by random 3 databases by qPCR in HCT116 and DLD1 cells transfected with siRNAs as indicated.

Supplementary Figure 3 The detection of GSDME/caspase-3 in MDA-MB-453 and normal human cells treated with siRNA targeting lncRNA AA388235. **(A)** Microscopic images of MDA-MB-453 cells transfected with siRNA as indicated. Red arrowheads indicated the pyroptotic cells and yellow arrowheads indicated the apoptosis cells. Scale bar, 100μm. **(B)** Immunoblotting assay of MDA-MB-453 cells transfected with siRNA as indicated. GSDME-FL, full-length of GSDME; GSDME-N, the N-terminal cleavage of GSDME; Cleaved-CAS3, the cleavage of Caspase3 p19/p17; Cleaved-PARP, the cleavage of PARP. **(C)** Comparison of LDH release-based cell death in MDA-MB-453 cells transfected with the siRNA as indicated. *P* value was calculated by one-way ANOVA. Mean ± SEM, ***P < 0.001, ****P < 0.0001. **(D, F, H, J)** Microscopic images of FHC, MCF12A, HEK293, and NCM460 cells transfected with siRNA as indicated. Red arrowheads indicated the pyroptotic cells, and yellow arrowheads indicated the apoptosis cells. Scale bar, 100μm. **(E, G, I, K)** Immunoblotting assay of FHC, MCF12A, HEK293, and NCM460 cells transfected with siRNA as indicated. si-2, siAA388235-2; si-6, siAA388235-6. GSDME-FL, full-length of GSDME; GSDME-N, the N-terminal cleavage of GSDME; Cleaved-CAS3, the cleavage of caspase-3 p19/p17; cleaved-PARP, the cleavage of PARP.

Supplementary Figure 4 siRNAs targeting mouse-specific lncRNA AA388235 have no effect on the death of mouse colorectal cancer cells. **(A, B)** Microscopic images of MC38 and CT26 cells transfected with siRNA as indicated. Scale bar, 100 μm. **(C, D)** Immunoblotting assay of MC38 and CT26 cells transfected with siRNA as indicated. Gsdme-FL, full-length of Gsdme; Cleaved-CAS3, the cleavage of caspase-3 p19/p17; cleaved-Parp, the cleavage of Parp. **(E)** Microscopic images of MC38 co-transfected with plasmid and siRNA as indicated. Red arrowheads indicated the pyroptotic cells. Scale bar, 100 μm. **(F)** The numbers of the pyroptotic cells of MC38 co-transfected with Gsdme and siRNA as indicated were calculated in randomly select five fields of view (×200). *P* value was calculated by one-way ANOVA. **(G)** Immunoblotting assay of MC38 co-transfected with Gsdme and siRNA as indicated. Gsdme-FL, full-length of Gsdme; Gsdme-N, the N-terminal cleavage products of Gsdme; Cleaved-CAS3, the cleavage of caspase-3 p19/p17; cleaved-Parp, the cleavage of Parp. Mean ± SEM, “ns” indicates no significance.

Supplementary Figure 5 Multiple-species conversation comparisons with mouse specific LncRNAs. **(A–G)** Graphical views showing multiple-species conversation comparisons with mouse specific LncRNAs using UCSC genome browser. The conservation scores were indicated by the gray peaks. This result showed that the seven randomly selected mouse LncRNA including GM11815 **(A)**, BC026762 **(B)**, GM15298 **(C)**, GM19792 **(D)**, GM14951 **(E)**, GM11851 **(F)** and GM15758 **(G)** only express in mouse.

Supplementary Figure 6 Multiple siRNAs targeting different mouse-specific lncRNAs induce cleavage of GSDME, caspase-3 and PARP. Immunoblotting assay of HCT116 and DLD1 cells transfected with siRNA as indicated. GSDME-FL, full-length of GSDME; GSDME-N, the N-terminal cleavage of GSDME; Cleaved-CAS3, the cleavage of caspase-3 p19/p17; cleaved-PARP, the cleavage of PARP. **(A)** Transfection with si-GM11815-1/2/3 induced the cleavage of GSDME, caspase-3 and PARP in HCT116. **(B)** Transfection with si-BC026762-1/2 induced the cleavage of GSDME, caspase-3 and PARP, and si-BC026762-3 had no effect in HCT116. **(C)** Transfection with si-GM15298-1/2/3 induced the cleavage of GSDME, caspase-3 and PARP in HCT116. **(D)** Transfection with si-GM19792-1/2 induced the cleavage of GSDME, caspase-3, and PARP, and si-GM19792-3 had no effect. **(E)** Transfection with si-GM14951-1 had no effect, and si-GM14951-2/3 induced the cleavage of GSDME, caspase-3 and PARP in HCT116. **(F)** Transfection with si-GM11851 and si-GM15758 had no effect on the cleavage of GSDME, caspase-3 and PARP in HCT116. **(G)** Transfection with si-GM11815-1/2/3 induced the cleavage of caspase-3 and PARP in DLD1. **(H)** Transfection with si-BC026762-1/2 induced the cleavage of caspase-3 and PARP in DLD1, and si-BC026762-3 had no effect. **(I)** Transfection with si-GM15298-1/2/3 had no effect on the cleavage of caspase-3 and PARP in DLD1. **(J)** Transfection with si-GM19792-1/2/3 had no effect on the cleavage of caspase-3 and PARP in DLD1. **(K)** Transfection with si-GM14951-1/2/3 induced the cleavage of caspase-3 and PARP in DLD1. **(L)** Transfection with si-GM11851 and si-GM15758 had no effect on the cleavage of caspase-3 and PARP in DLD1.

Supplementary Figure 7 The detection of AA388235 level by qPCR in different transfection as indicated. **(A, B)** The level of AA388235 in HCT116 and DLD1 cells transfected with plasmid as indicated. *P* value was calculated by Student’s t test. **(C, D)** The level of AA388235 in MC38 and CT26 cells transfected with plasmid as indicated. *P* value was calculated by Student’s t test. **(E, F)** The level of AA388235 in HCT116 and DLD1 cells co-transfected with plasmid and siRNA as indicated. *P* value was calculated by two-way ANOVA. Mean ± SEM, ***P* < 0.01, ****P* < 0.001, *****P* < 0.0001.
