# Supplementary figures and images for: siRNAs Targeting Mouse-Specific lncRNA AA388235 Induce Human Tumor Cell Pyroptosis/Apoptosis
Source: Front Oncol. 2021 Jun 14;11:662444. doi: 10.3389/fonc.2021.662444 (PMC8236890; doi:10.3389/fonc.2021.662444)

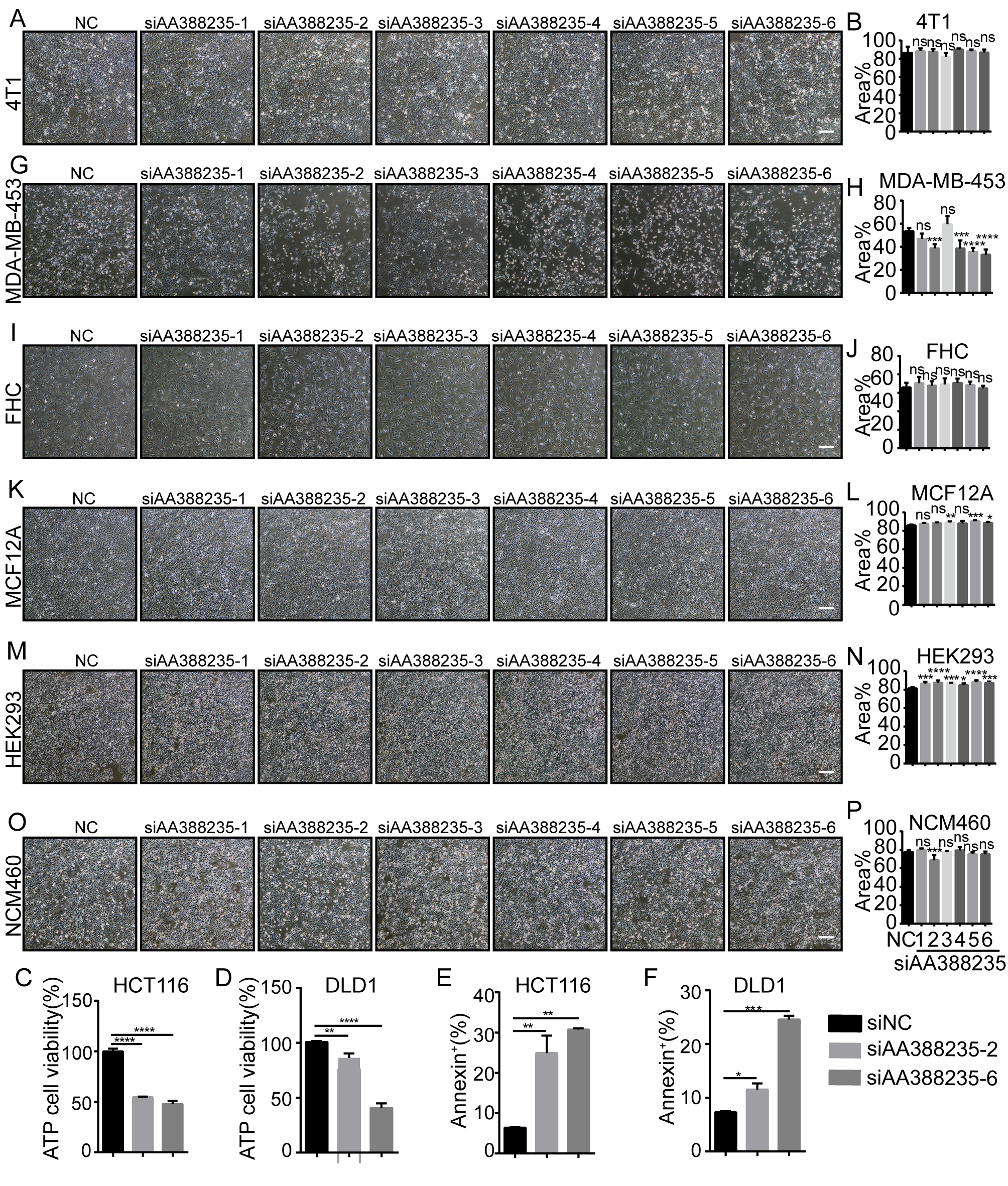

Supplement: Supplementary Figure 1 — siRNAs targeting mouse-specific lncRNA AA388235 have no/little effect on 4T1 and human normal cells but induce human tumor cells death. (A, B) 4T1 cells were seeded and treated as indicated. After 48 h, the static bright-field cell images were captured (A). Then the dead cells were washed off with PBS, and the living cells were captured to measure the area percentage of survival cells using Image J software from five independent perspectives (B).Scale bar, 200μm. P value was calculated by one-way ANOVA. (C, D) Comparison of ATP cell viability in HCT116 and DLD1 cells transfected with the siRNAs as indicated. P value was calculated by one-way ANOVA. (E, F) Flow cytometry of PI (propidium iodide) and Annexin V-fluorescein isothiocyanate (FITC)-stained cells. The percentage of Annexin+ cells is the sum of PI+/Annexin V+ and PI−/Annexin V+ cells. P value was calculated by one-way ANOVA. (G, H) MDA-MB-453 cells were seeded and treated as indicated. After 48 h, the static bright-field cell images were captured (G). Then the dead cells were washed off with PBS, and the living cells were captured to measure the area percentage of survival cells using Image J software from five independent perspectives (H).Scale bar, 200μm. P value was calculated by one-way ANOVA. (I–P) Cells were seeded and treated as indicated. After 48 h, the static bright-field cell images were captured (I, K, M, O). Then the dead cells were washed off with PBS, and the living cells were captured to measure the area percentage of survival cells using Image J software from five independent perspectives (J, L, N, P). Scale bar, 200μm. P value was calculated by one-way ANOVA. Mean ± SEM, *P < 0.05, **P < 0.01, ***P < 0.001, ****P < 0.0001, “ns” indicates no significance. [file Image_1.tif]

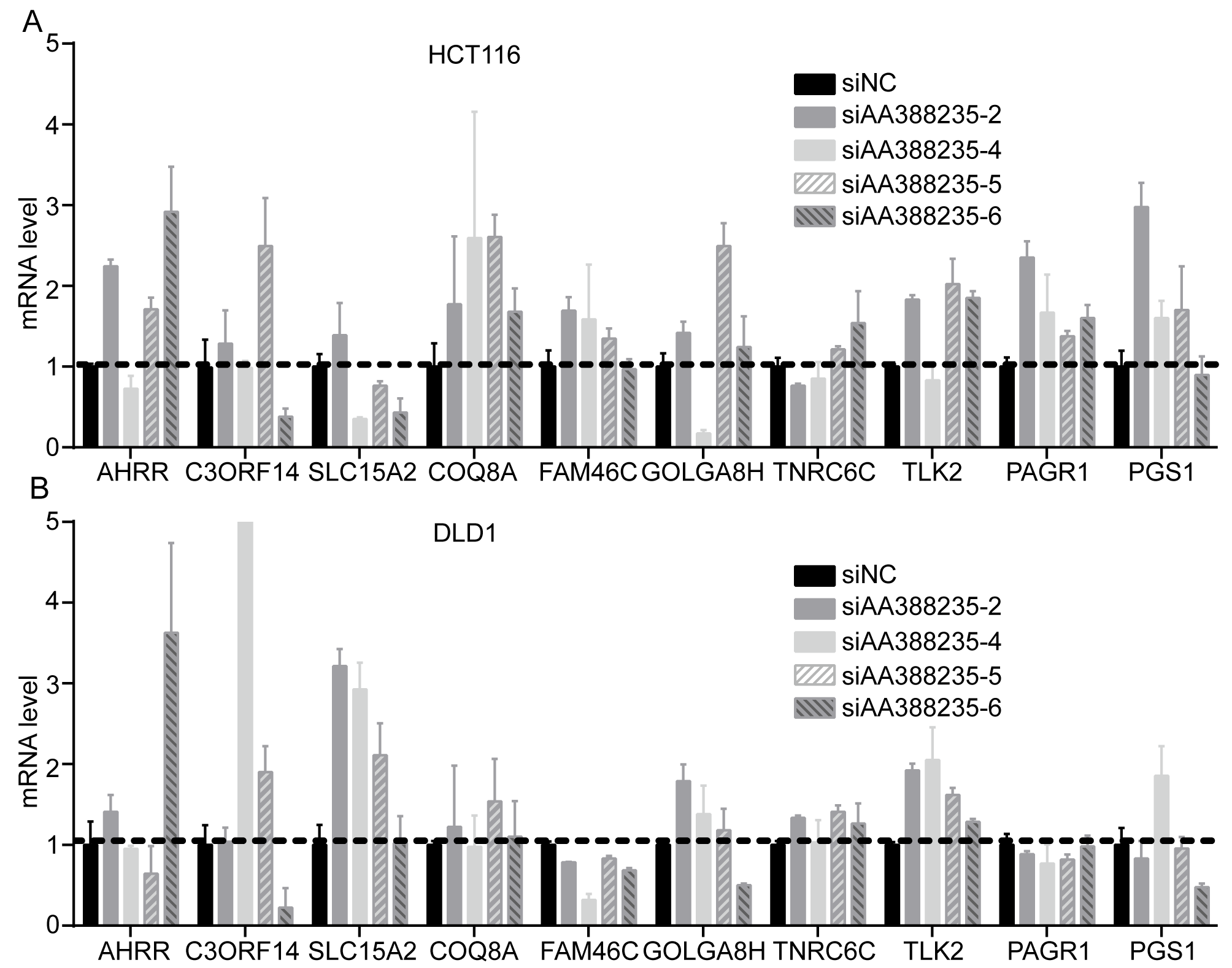

Supplement: Supplementary Figure 2 — siRNAs targeting mouse-specific lncRNA AA388235 do not target one common human gene. (A, B) The detection of predicted common off-target gene between the siAA388235-2,4,5, and 6 recognized by random 3 databases by qPCR in HCT116 and DLD1 cells transfected with siRNAs as indicated. [file Image_2.tif]

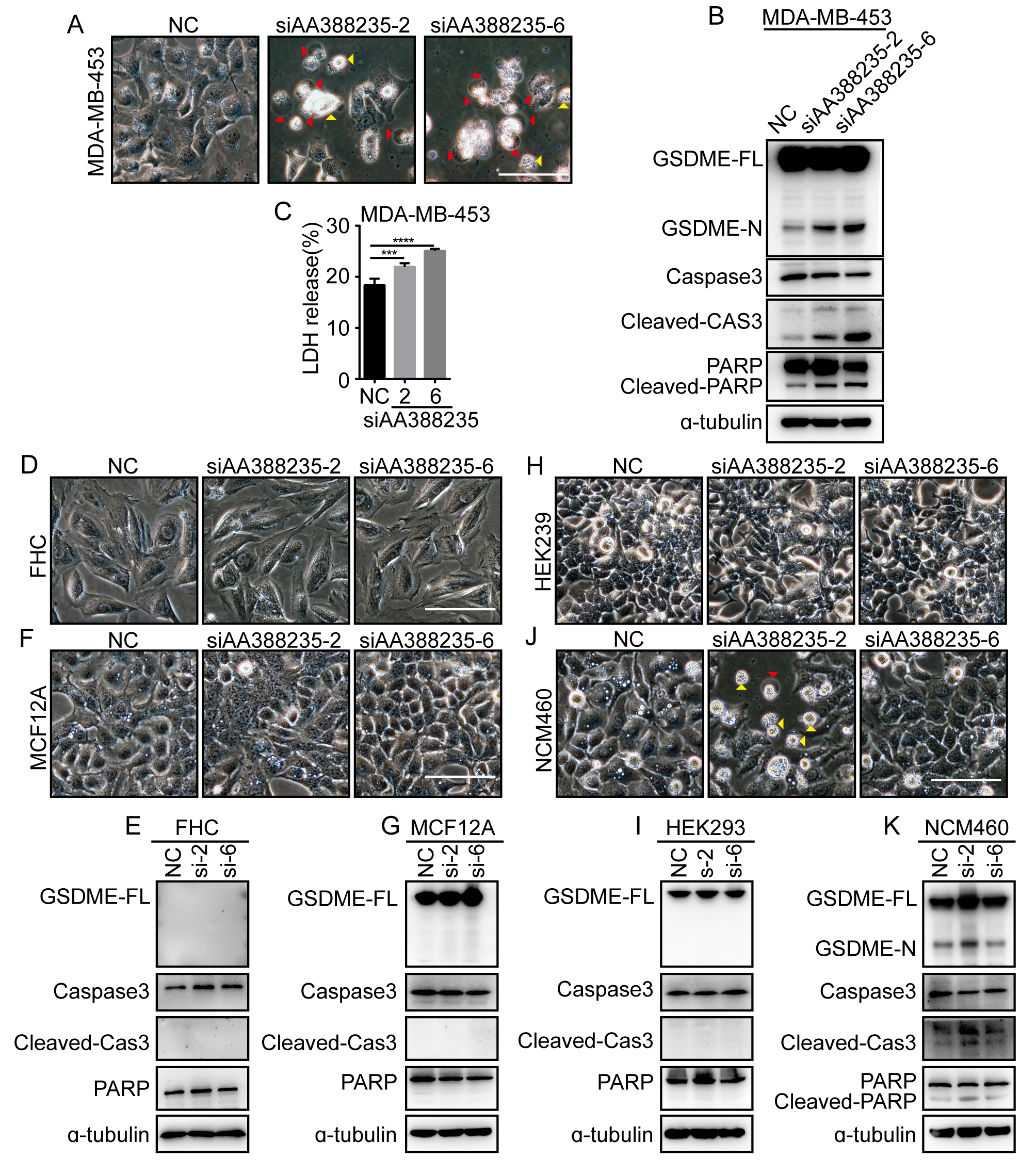

Supplement: Supplementary Figure 3 — The detection of GSDME/caspase-3 in MDA-MB-453 and normal human cells treated with siRNA targeting lncRNA AA388235. (A) Microscopic images of MDA-MB-453 cells transfected with siRNA as indicated. Red arrowheads indicated the pyroptotic cells and yellow arrowheads indicated the apoptosis cells. Scale bar, 100μm. (B) Immunoblotting assay of MDA-MB-453 cells transfected with siRNA as indicated. GSDME-FL, full-length of GSDME; GSDME-N, the N-terminal cleavage of GSDME; Cleaved-CAS3, the cleavage of Caspase3 p19/p17; Cleaved-PARP, the cleavage of PARP. (C) Comparison of LDH release-based cell death in MDA-MB-453 cells transfected with the siRNA as indicated. P value was calculated by one-way ANOVA. Mean ± SEM, ***P < 0.001, ****P < 0.0001. (D, F, H, J) Microscopic images of FHC, MCF12A, HEK293, and NCM460 cells transfected with siRNA as indicated. Red arrowheads indicated the pyroptotic cells, and yellow arrowheads indicated the apoptosis cells. Scale bar, 100μm. (E, G, I, K) Immunoblotting assay of FHC, MCF12A, HEK293, and NCM460 cells transfected with siRNA as indicated. si-2, siAA388235-2; si-6, siAA388235-6. GSDME-FL, full-length of GSDME; GSDME-N, the N-terminal cleavage of GSDME; Cleaved-CAS3, the cleavage of caspase-3 p19/p17; cleaved-PARP, the cleavage of PARP. [file Image_3.tif]

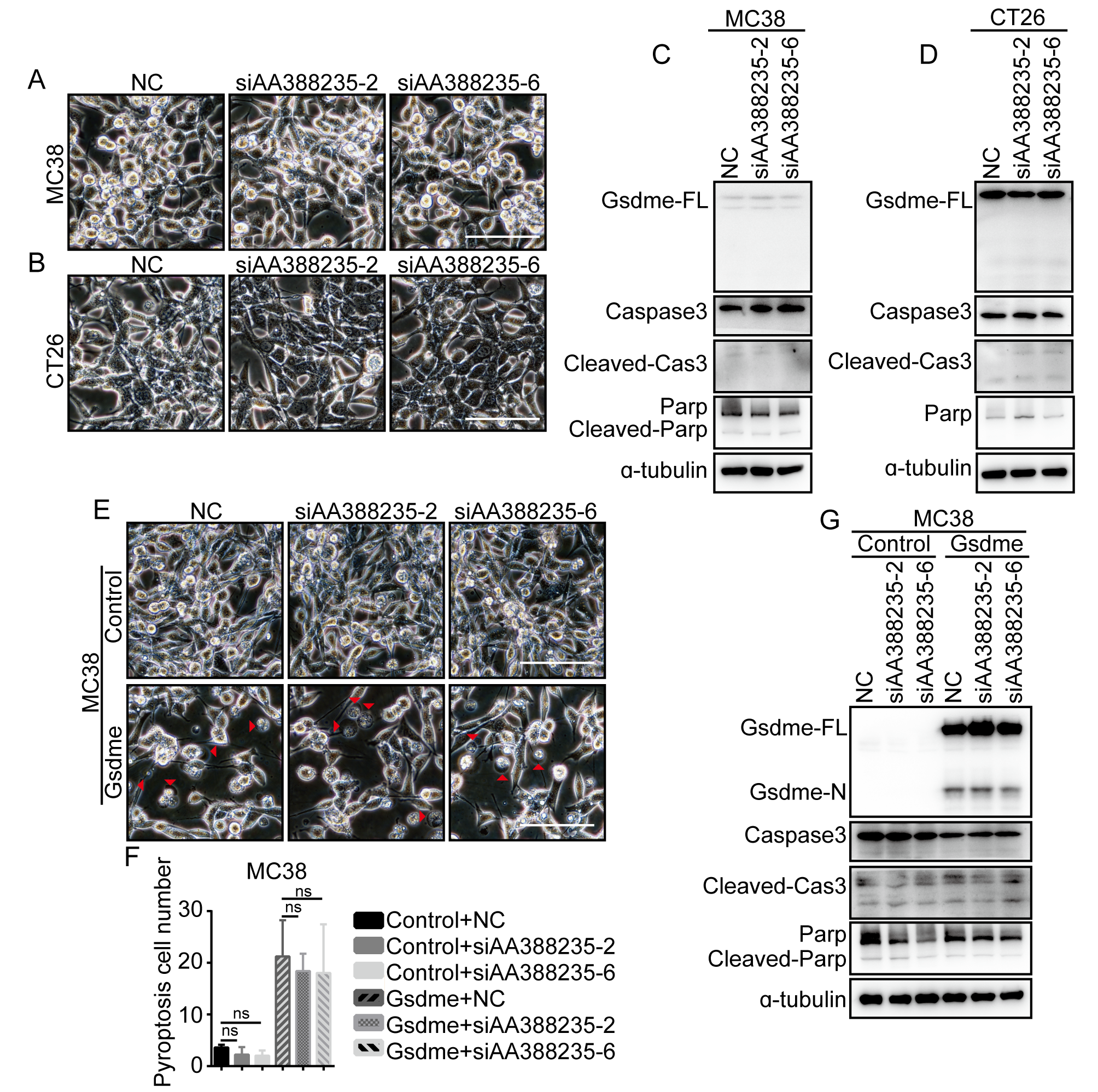

Supplement: Supplementary Figure 4 — siRNAs targeting mouse-specific lncRNA AA388235 have no effect on the death of mouse colorectal cancer cells. (A, B) Microscopic images of MC38 and CT26 cells transfected with siRNA as indicated. Scale bar, 100 μm. (C, D) Immunoblotting assay of MC38 and CT26 cells transfected with siRNA as indicated. Gsdme-FL, full-length of Gsdme; Cleaved-CAS3, the cleavage of caspase-3 p19/p17; cleaved-Parp, the cleavage of Parp. (E) Microscopic images of MC38 co-transfected with plasmid and siRNA as indicated. Red arrowheads indicated the pyroptotic cells. Scale bar, 100 μm. (F) The numbers of the pyroptotic cells of MC38 co-transfected with Gsdme and siRNA as indicated were calculated in randomly select five fields of view (×200). P value was calculated by one-way ANOVA. (G) Immunoblotting assay of MC38 co-transfected with Gsdme and siRNA as indicated. Gsdme-FL, full-length of Gsdme; Gsdme-N, the N-terminal cleavage products of Gsdme; Cleaved-CAS3, the cleavage of caspase-3 p19/p17; cleaved-Parp, the cleavage of Parp. Mean ± SEM, “ns” indicates no significance. [file Image_4.tif]

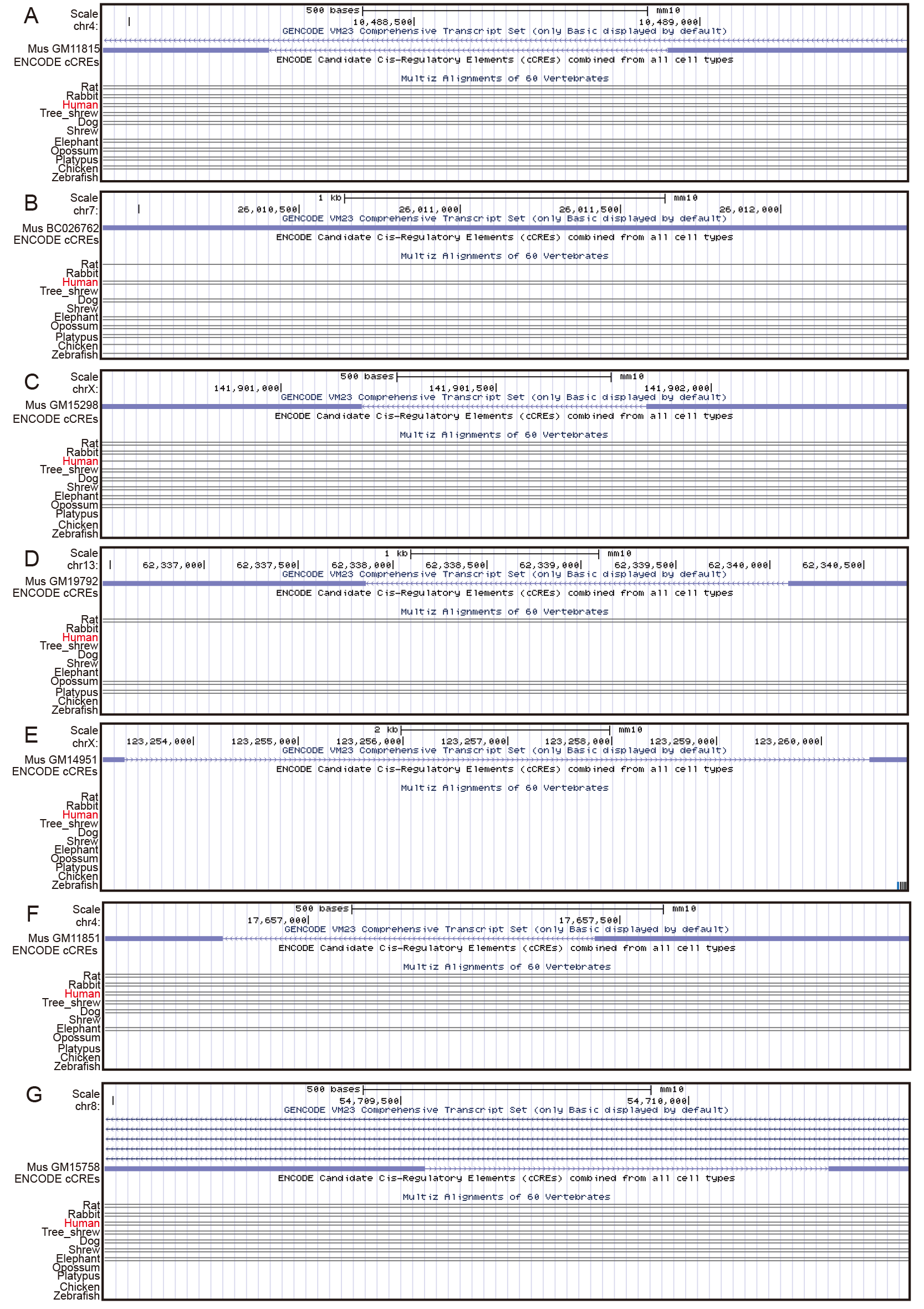

Supplement: Supplementary Figure 5 — Multiple-species conversation comparisons with mouse specific LncRNAs. (A–G) Graphical views showing multiple-species conversation comparisons with mouse specific LncRNAs using UCSC genome browser. The conservation scores were indicated by the gray peaks. This result showed that the seven randomly selected mouse LncRNA including GM11815 (A), BC026762 (B), GM15298 (C), GM19792 (D), GM14951 (E), GM11851 (F) and GM15758 (G) only express in mouse. [file Image_5.tif]

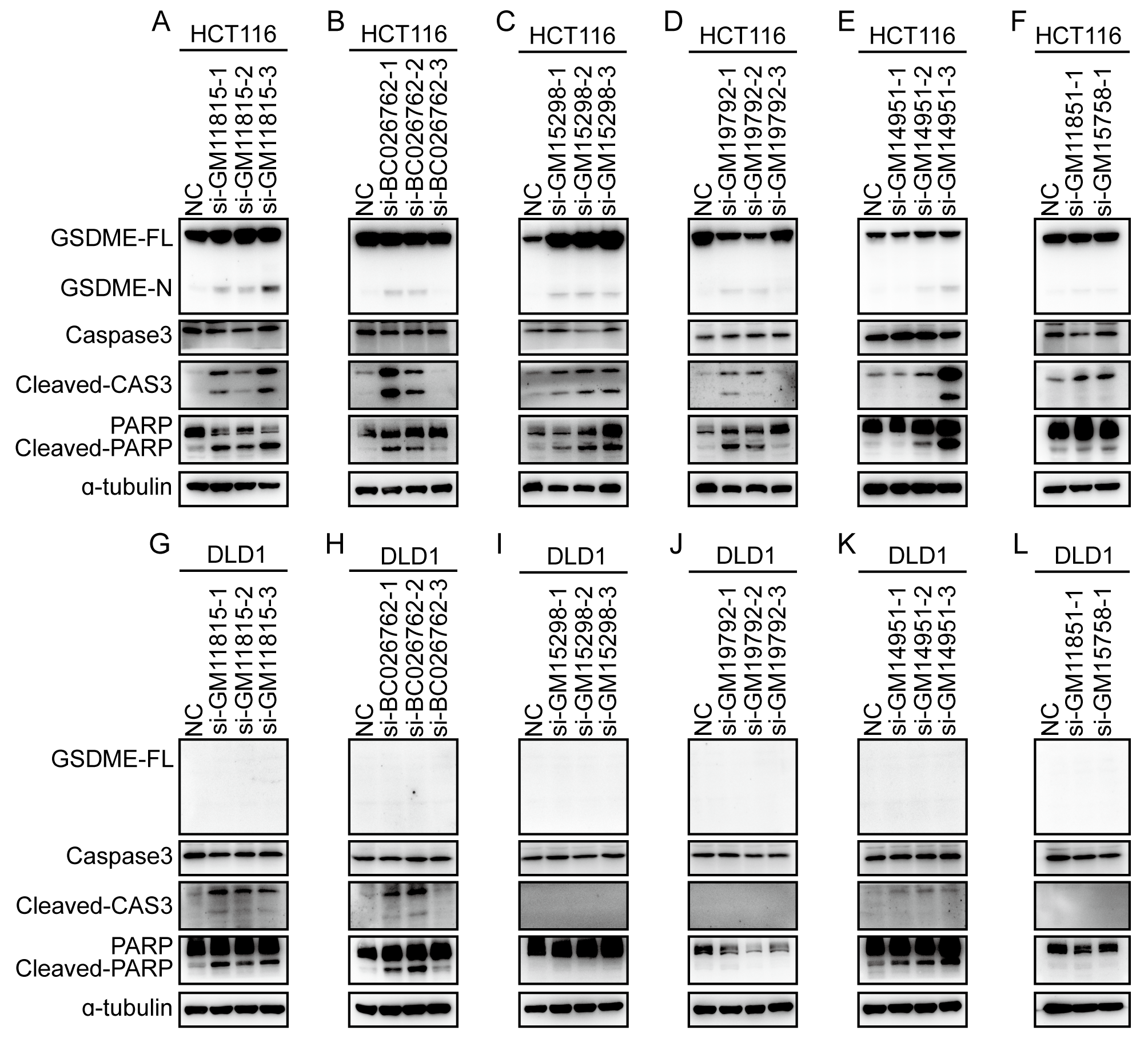

Supplement: Supplementary Figure 6 — Multiple siRNAs targeting different mouse-specific lncRNAs induce cleavage of GSDME, caspase-3 and PARP. Immunoblotting assay of HCT116 and DLD1 cells transfected with siRNA as indicated. GSDME-FL, full-length of GSDME; GSDME-N, the N-terminal cleavage of GSDME; Cleaved-CAS3, the cleavage of caspase-3 p19/p17; cleaved-PARP, the cleavage of PARP. (A) Transfection with si-GM11815-1/2/3 induced the cleavage of GSDME, caspase-3 and PARP in HCT116. (B) Transfection with si-BC026762-1/2 induced the cleavage of GSDME, caspase-3 and PARP, and si-BC026762-3 had no effect in HCT116. (C) Transfection with si-GM15298-1/2/3 induced the cleavage of GSDME, caspase-3 and PARP in HCT116. (D) Transfection with si-GM19792-1/2 induced the cleavage of GSDME, caspase-3, and PARP, and si-GM19792-3 had no effect. (E) Transfection with si-GM14951-1 had no effect, and si-GM14951-2/3 induced the cleavage of GSDME, caspase-3 and PARP in HCT116. (F) Transfection with si-GM11851 and si-GM15758 had no effect on the cleavage of GSDME, caspase-3 and PARP in HCT116. (G) Transfection with si-GM11815-1/2/3 induced the cleavage of caspase-3 and PARP in DLD1. (H) Transfection with si-BC026762-1/2 induced the cleavage of caspase-3 and PARP in DLD1, and si-BC026762-3 had no effect. (I) Transfection with si-GM15298-1/2/3 had no effect on the cleavage of caspase-3 and PARP in DLD1. (J) Transfection with si-GM19792-1/2/3 had no effect on the cleavage of caspase-3 and PARP in DLD1. (K) Transfection with si-GM14951-1/2/3 induced the cleavage of caspase-3 and PARP in DLD1. (L) Transfection with si-GM11851 and si-GM15758 had no effect on the cleavage of caspase-3 and PARP in DLD1. [file Image_6.tif]

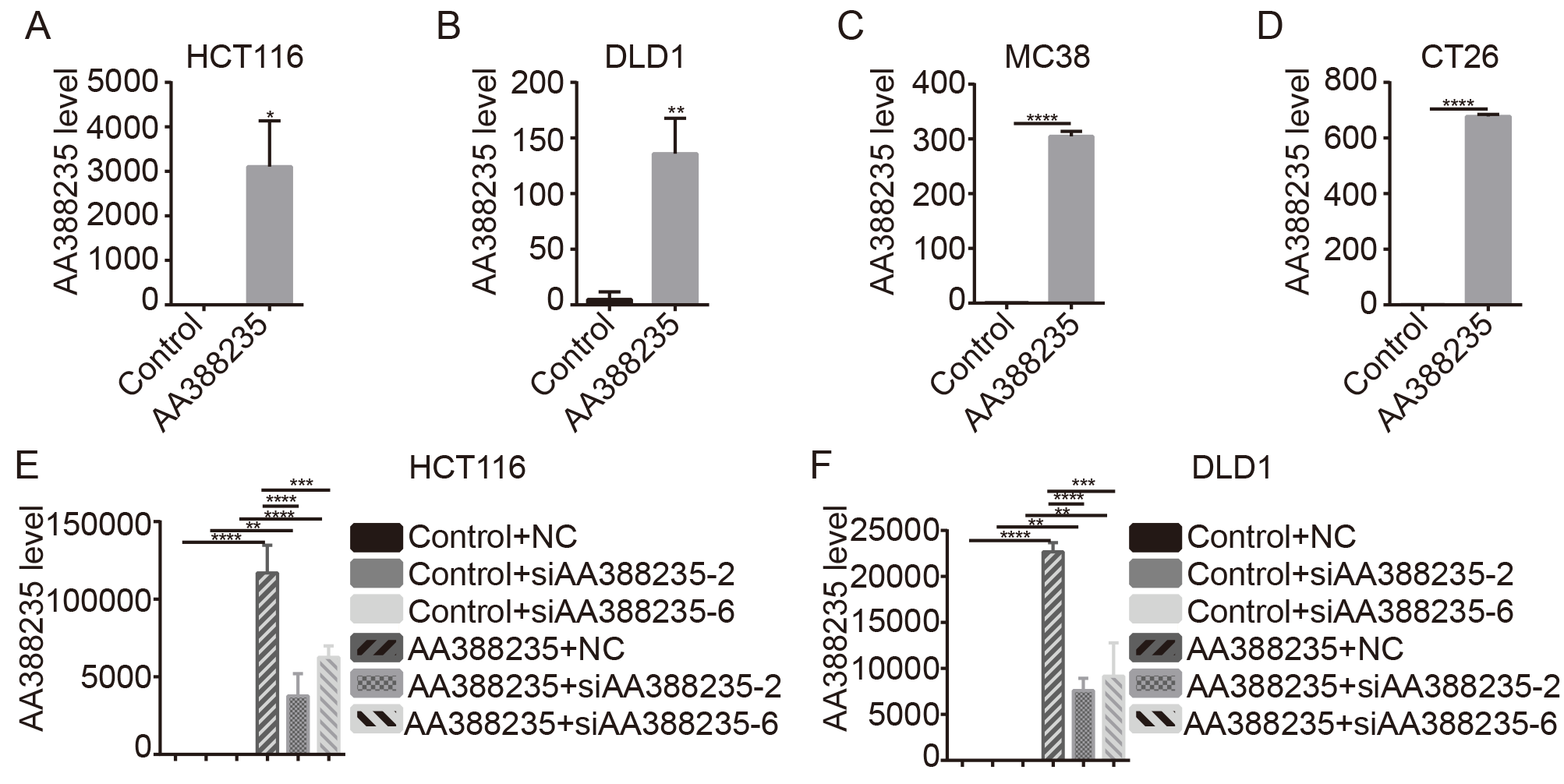

Supplement: Supplementary Figure 7 — The detection of AA388235 level by qPCR in different transfection as indicated. (A, B) The level of AA388235 in HCT116 and DLD1 cells transfected with plasmid as indicated. P value was calculated by Student’s t test. (C, D) The level of AA388235 in MC38 and CT26 cells transfected with plasmid as indicated. P value was calculated by Student’s t test. (E, F) The level of AA388235 in HCT116 and DLD1 cells co-transfected with plasmid and siRNA as indicated. P value was calculated by two-way ANOVA. Mean ± SEM, **P < 0.01, ***P < 0.001, ****P < 0.0001. [file Image_7.tif]
