# Supplementary material for: siRNAs Targeting Mouse-Specific lncRNA AA388235 Induce Human Tumor Cell Pyroptosis/Apoptosis
Source: Front Oncol. 2021 Jun 14;11:662444. doi: 10.3389/fonc.2021.662444 (PMC8236890; doi:10.3389/fonc.2021.662444)
Supplement: Supplementary file 8 [file Table_1.docx]

Table S1. The sequence of siRNA and siDNA.

| Name | Sequence |
| --- | --- |
| si-AA388235-1(dsRNA) | Sense:5'-CCAUAAAGAUGUAAGCAAUdTdT-3' |
| si-AA388235-2(dsRNA) | Sense:5'-GAAGGAAGUUAUAGCUUUAdTdT-3' |
| si-AA388235-3(dsRNA) | Sense:5'-GGAAGCAGAGACAACAUAAdTdT-3' |
| si-AA388235-4(dsRNA) | Sense:5'-GCACAUACAUAUUCACAUAdTdT-3' |
| si-AA388235-5(dsRNA) | Sense:5'-CCCACUCAACAACCACAUAdTdT-3' |
| si-AA388235-6(dsRNA) | Sense:5'-CCAUUCUUGGCCUUCUUCAdTdT-3' |
| si-AA388235-2-guide(ssRNA) | 5'-GAAGGAAGUUAUAGCUUUAdTdT-3' |
| si-AA388235-2-dsDNA(dsDNA) | Sense:5'-GAAGGAAGTTATAGCTTTAdTdT-3' |
| si-AA388235-6-guide(ssRNA) | 5'-UGAAGAAGGCCAAGAAUGGdTdT-3' |
| si-AA388235-6-passager(ssRNA) | 5'-CCAUUCUUGGCCUUCUUCAdTdT-3' |
| si-AA388235-6-dsDNA(dsDNA) | Sense:5'-CCATTCTTGGCCTTCTTCAdTdT-3' |
| si-Gm11815-1(dsRNA) | Sense:5'-CUGGGAAGGAGCGGUUAAUdTdT-3' |
| si-Gm11815-2(dsRNA) | Sense:5'-CUGACUAUCUAUCCCUUGCdTdT-3' |
| si-Gm11815-3(dsRNA) | Sense:5'-CCAGGAUAAGGGACAAGAAdTdT-3' |
| si-BC026762-1(dsRNA) | Sense:5'-GAUGUUGCCUAGAGUUUCAdTdT-3' |
| si-BC026762-2(dsRNA) | Sense:5'-CCAUAGGGUUGUAAAGCUAdTdT-3' |
| si-BC026762-3(dsRNA) | Sense:5'-CAGGCAGUAUUAUCCAUGUdTdT-3' |
| si-Gm15298-1(dsRNA) | Sense:5'-UCGAGAGUUGGUGCUGAGAdTdT-3' |
| si-Gm15298-2(dsRNA) | Sense:5'-CUCUGGCUAUCUAUCCCUUdTdT-3' |
| si-Gm15298-3(dsRNA) | Sense:5'-CACUGGGAAGGAGCCGUUAdTdT-3' |
| si-Gm19792-1(dsRNA) | Sense:5'-GCUGCUAUCUUCAUUCCCAdTdT-3' |
| si-Gm19792-2(dsRNA) | Sense:5'-CAGCACCCACGGGAGAAAUdTdT-3' |
| si-Gm19792-3(dsRNA) | Sense:5'-CAUACUGGUUCGUGAUCAUdTdT-3' |
| si-Gm14951-1(dsRNA) | Sense:5'-GCAUGGACGUGUAAAUAUAdTdT-3' |
| si-Gm14951-2(dsRNA) | Sense:5'-CUAGGCAUGUAUAGAUUAAdTdT-3' |
| si-Gm14951-3(dsRNA) | Sense:5'-GUUGAAAUAUGGUGGACAAdTdT-3' |
| si-Gm11851(dsRNA) | Sense:5'-GAGAGAUGAAGACUGAAUAdTdT-3' |
| si-Gm15758(dsRNA) | Sense:5'-CGCUGCUACAGGAAGGAUUdTdT-3' |

dsRNA:double strand RNA ;ssRNA:single strand RNA;dsDNA:double strand DNA

Table S2. The primers of selected genes for real time PCR.

| Gene | Forward primer (5’-3’) | Reverse primer (5’-3’) |
| --- | --- | --- |
| AA388235 | TTGCTCTGTGTCTGCTTGCTCTTC | CAGGTACAGTCCAGTCCTCTCAGTAG |
| GSDMA | CAACAGACACTGGGAATTTTGG | CTTCACCGTCTTTGGTACATCC |
| GSDMB | ACATGGAGGACCCAGACAAG | CACAGAGAATTCGTGCCTCA |
| GSDMC | TCAGAGACAGAGGGGCTCTACA | GTTGGAAGTCACTCAGCACCAT |
| GSDMD | ACAGCTCCAGCACCTCAATGAATG | GCACCTCAGTCACCACGTACAC |
| AHRR | GAGACAGGAGATGATGCTATCC | TTTTCCTTGAAACTGCATCGTC |
| C3ORF14 | CAACTCCAAACTGTTGAGACTG | TCCTGGTCTGTAGTGACTTTTC |
| SLC15A2 | CATTTATCACACCCATGCTGAG | AGAACGGTTCTTGAAACGATTG |
| COQ8A | ATGAAAACTCTCAACAACGACC | GGTTGTTGACATCACTGTTGAT |
| FAM46C | CAGGCATCAAAGTGCACGAC | AGCTTGTTCACACCCTCTGG |
| GOLGA8H | CTCTGCTTCATCCACCACTG | AGCCGCAATACCATCTGC |
| TNRC6C | TAATCCAGAACAGTGAGTCACC | GTTGATGCTAGAGCCATTTGAG |
| TLK2 | GACCGCTTGAGACTGGGCCACT | AATGAGGCTCCGTGTCGGACAG |
| PAGR1 | AAGAGGAAGAGGAAAAACCACA | GCTTCTTGTGTCTCTTCATGTC |
| PGS1 | GACAGTTCTTCAGTGAGGTGT | TGAACTGACCTGTACCCAAAAT |
| GAPDH | GACTCATGACCACAGTCCATGC | AGAGGCAGGGATGATGTTCTG |
| Gapdh | GCCTGGAGAAACCTGCCAAGTATG | GAGTGGGAGTTGCTGTTGAAGTCG |
